# Stability of hypothetical Ag^II^Cl_2_ polymorphs under high pressure, revisited: a computational study

**DOI:** 10.1038/s41598-022-05211-0

**Published:** 2022-01-21

**Authors:** Adam Grzelak, Wojciech Grochala

**Affiliations:** Center for New Technologies, University of Warsaw, Banacha 2C, 02-097 Warszawa, Poland

**Keywords:** Computational chemistry, Density functional theory, Solid-state chemistry, Structure prediction

## Abstract

A comparative computational study of stability of candidate structures for an as-yet unknown silver dichloride AgCl_2_ is presented. It is found that all considered candidates have a negative enthalpy of formation, but are unstable towards charge transfer and decomposition into silver(I) chloride and chlorine within the DFT and hybrid-DFT approaches in the entire studied pressure range. Within SCAN approach, several of the “true” Ag^II^Cl_2_ polymorphs (i.e. containing Ag(II) species) exhibit a region of stability below ca. 20 GPa. However, their stability with respect to aforementioned decomposition decreases with pressure by account of all three DFT methods, which suggests a limited possibility of high-pressure synthesis of AgCl_2_. Some common patterns in pressure-induced structural transitions observed in the studied systems also emerge, which further testify to an instability of hypothetical AgCl_2_ towards charge transfer and phase separation.

## Introduction

Chemistry of silver(II) compounds constitutes a topic of studies that is both demanding—particularly due to the extremely strong oxidizing properties of these compounds—as well as interesting, as evidenced by the body of works discussing them in relation to oxocuprates—a well-known family of precursors for high-pressure superconductors^1,2^. Inspired by experimental works exploring high-pressure phase transitions of AgF_2_^3,4^, as well as most recent computational study of thermodynamic stability of hypothetical mixed-valence silver fluorides (including at elevated pressure conditions)^5^, this work is a continuation to a previous systematic study, which explored relative stability of multiple hypothetical polymorphs of Ag^II^Cl_2_—an as-yet unknown analogue of AgF_2_^6^. The aforementioned study found that a true silver(II) chloride is likely to be unstable towards charge transfer and phase separation into AgCl and Cl_2_ at ambient pressure conditions. This work aims to extend these considerations into high-pressure regime, in the hope that applying extreme conditions could stabilize Ag^II^Cl_2_. In particular, the previous study found that a layered, AgF_2_-type polymorph of AgCl_2_ could be stabilized at a pressure of ca. 35 GPa, due to relatively low molar volume^6^.

The interest in this particular compound stems from its potential similarity to AgF_2_, which has recently been shown to be an excellent analog of oxocuprates in terms of structure and very strong magnetic interactions^2^. In fact, Ag^II^Cl_2_—if obtained, and providing a suitable structural arrangement—could be expected to host even stronger antiferromagnetic superexchange than AgF_2_, due to stronger covalence of Ag–Cl bonding, as predicted from differences in electronegativity (Ag: 1.93, Cl: 3.16, F: 3.98—Pauling scale). Overall, this work is part of a joint computational and experimental effort: to synthesize Ag^II^Cl_2_ utilizing high pressure and high temperature experimental techniques, coupled with computational methods providing insight into understanding the expected products and phases. On top of that, the added value of studies in high-pressure regime is that the observed changes in structure and bonding induced by pressure can provide meaningful insight into the chemical nature of the studied compounds^7^.

## Results

### Stability of AgCl_2_ phases

Seven different candidate structures for polymorphs of AgCl_2_ were considered in this work. Six of them were derived from the previous, ambient-pressure study^6^. They were:

- AgF_2_ type (*Pbca*)—corrugated layers made up of [AgCl_4_] square subunits;
- CuCl_2_ type (*P*1)—1D chains made up of [AgCl_4_] square subunits;
- AuCl_2_ type (*P*-1)—a kind of nanotubular polymorph derived from 0D molecular structure of AuCl_2_ (see below);
- Ag(I)r type (*P*-1)—a structure composed of double layers of rocksalt-type AgCl interspersed with Cl_2_ bridges;
- Ag(I)h type (*P*-1)—similar as above, but with hexagonal double layers of AgCl;
- MnO_2_ type (*P*2_1_/*c*)—a different arrangement of corrugated layers made up of pairs of [AgCl_4_] square subunits.

**Figure 1:**
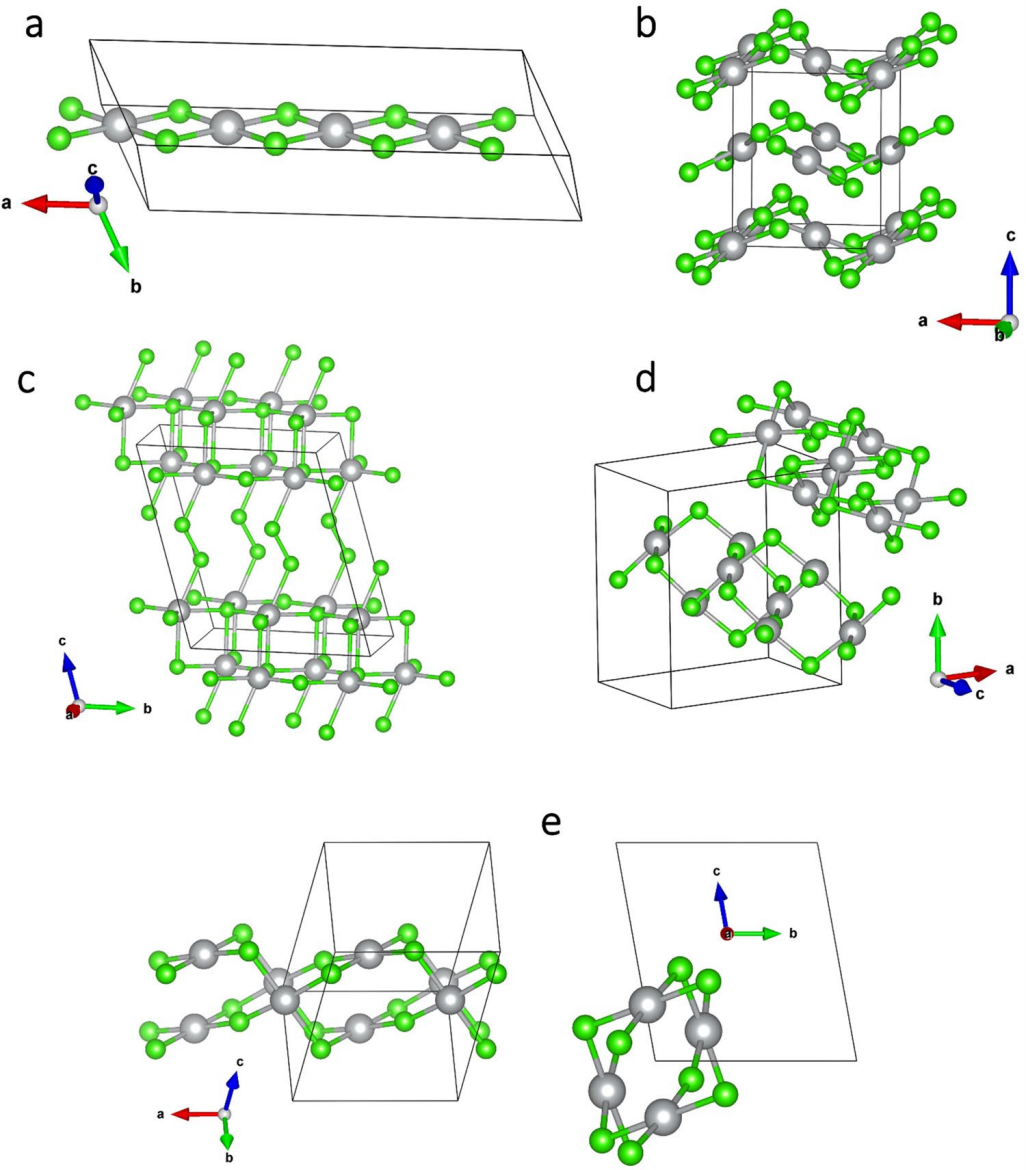
Selected structures of AgCl_2_ candidate polymorphs. **(a)** CuCl_2_-type, **(b)** AgF_2_-type, **(c)** Ag(I)r, **(d)** AgF_2_-HP-type, **(e)** AuCl_2_-type. Ag—grey, Cl—green.

Additionally, the high-pressure, nanotubular polymorph of AgF_2_ (referred to as HP-AgF_2_, space group *Pbcn*) was considered as a candidate^4^. Structures of selected polymorphs described above are presented in Fig. 1. Structures of all of the aforementioned candidate polymorphs are provided in Supplementary Information.

Importantly, Ag(I)r and Ag(I)h polymorphs do not contain Ag(II) species and instead are composed of sub-structures of Ag^I^Cl and Cl_2_ molecules. For the remaining five Ag^II^Cl_2_ polymorphs containing paramagnetic d^9^ silver cation, magnetic interactions were taken into account:

- AgF_2_ type: 2D antiferromagnetic (AFM) coupling within layers;
- CuCl_2_ type: AABB-type AFM coupling within chains, known to exist in CuCl_2_^8^, and found to be the lowest-energy magnetic solution for AgCl_2_ in this arrangement^6^;
- AgF_2_ HP type: magnetic dimers coupled along ~ 180 degrees Ag–F–Ag bridges within nanotubes;
- MnO_2_ type: AABB-type AFM coupling—ferromagnetic (FM) between adjacent [AgCl_4_] squares and AFM between pairs.

The AuCl_2_ type derives from a structure of gold(I,III) chloride, a mixed-valence compound which consists of Au_4_Cl_8_ molecules, each containing two Ag(I) and two Ag(III) species^9^. When this structure is taken as a starting point for geometry optimization, all three computational methods yield a nanotubular polymorph somewhat similar to the AgF_2_ HP type. However, only the HSE06 approach is able to reproduce the mixed-valence nature of AuCl_2_ and of the corresponding nanotubular polymorph of AgCl_2_ derived from the former (as evidenced by two different coordination patterns of Ag sites in that solution)^6^. On the other hand, in the PBEsol + U and SCAN calculations, a magnetic model with dimers as in the AgF_2_ HP type polymorphs was considered.

Stability of the studied candidate structures was evaluated using two parameters: (a) enthalpy of formation (labelled henceforth as ΔH_f_), according to a reaction:

1 $$Ag+{Cl}_{2}\to {AgCl}_{2}$$

and (b) stability towards decomposition into AgCl and Cl_2_ (ΔH_r_), or more precisely, the enthalpy of reaction:

2 $$AgCl+\frac{1}{2}{Cl}_{2}\to {AgCl}_{2}$$

**Figure 2:**
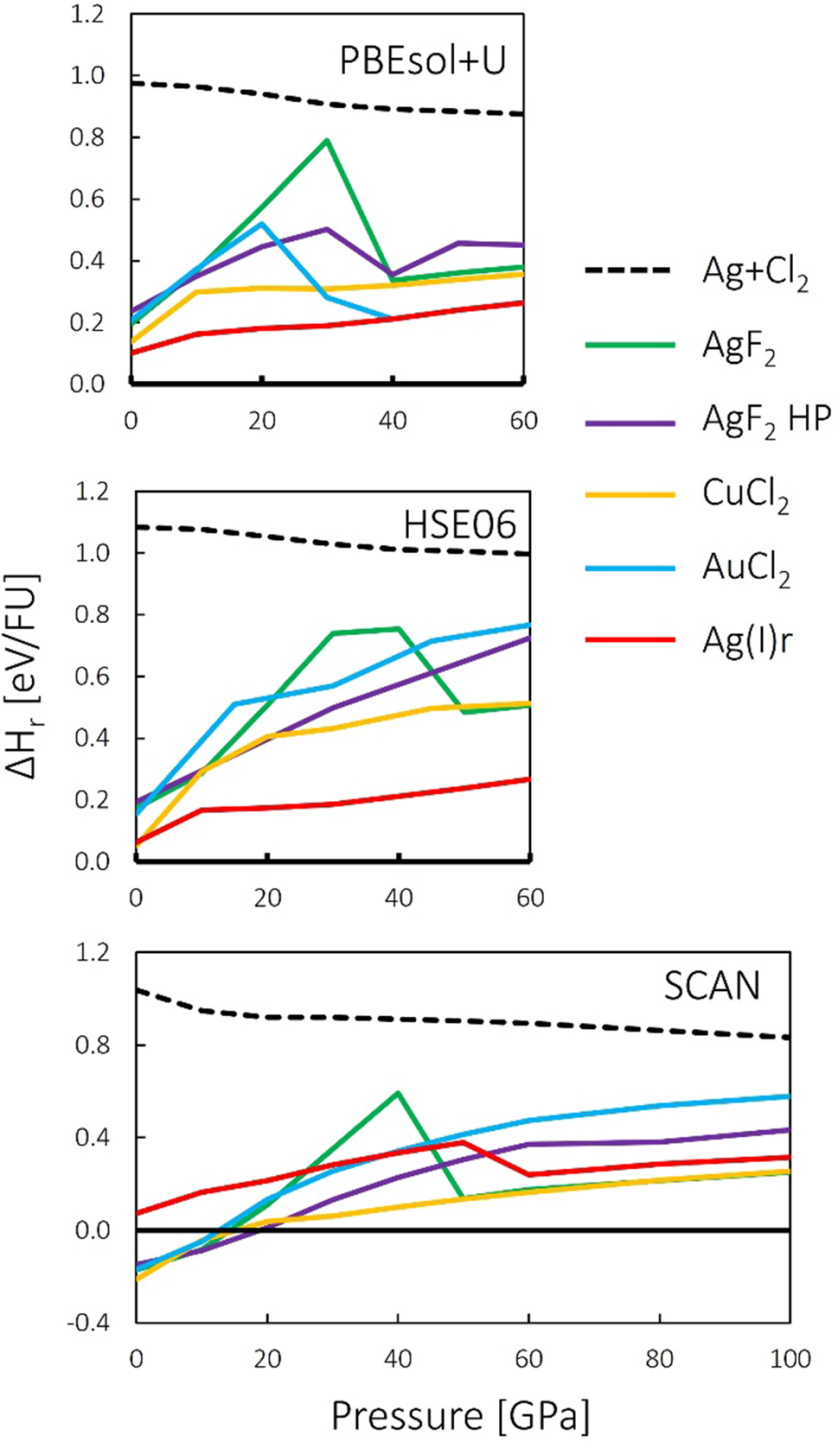
Stability of selected studied polymorphs of AgCl_2_, plotted as enthalpy per formula unit relative to AgCl + ½Cl_2_ (ΔH_r_). Top panel—PBEsol + U (GGA functional), middle panel—HSE06 (hybrid functional), bottom panel—SCAN (meta-GGA functional). *FU* formula unit.

Defined in this manner, both parameters indicate thermodynamic instability when positive. Figure 2 shows plots of stability of studied AgCl_2_ candidate types in terms of ΔH_r_ for all three methods used in this work. Enthalpy of Ag + Cl_2_ mixture relative to AgCl + ½Cl_2_ is also plotted for comparison.

The studied polymorphs exhibit negative (favorable) enthalpies of formation within the entire studied pressure range in all three computational approaches. This is indirectly visible in Fig. 2 as the fact that curves for those polymorphs lie below the curve for Ag + Cl_2_. On the other hand, they were found to be unstable in terms of ΔH_r_ in the entire studied pressure range within PBEsol + U and HSE06. However, results of SCAN calculations indicate moderate stability of Ag^II^Cl_2_ polymorphs below ca. 20 GPa. Ag(I)r polymorph is the most stable among AgCl_2_ candidate structures, according to PBEsol + U and HSE06 results, while this is not the case in SCAN picture. Given that it contains separate sub-phases of AgCl and Cl_2_, this further points to an instability towards charge transfer and phase separation.

**Figure 3:**
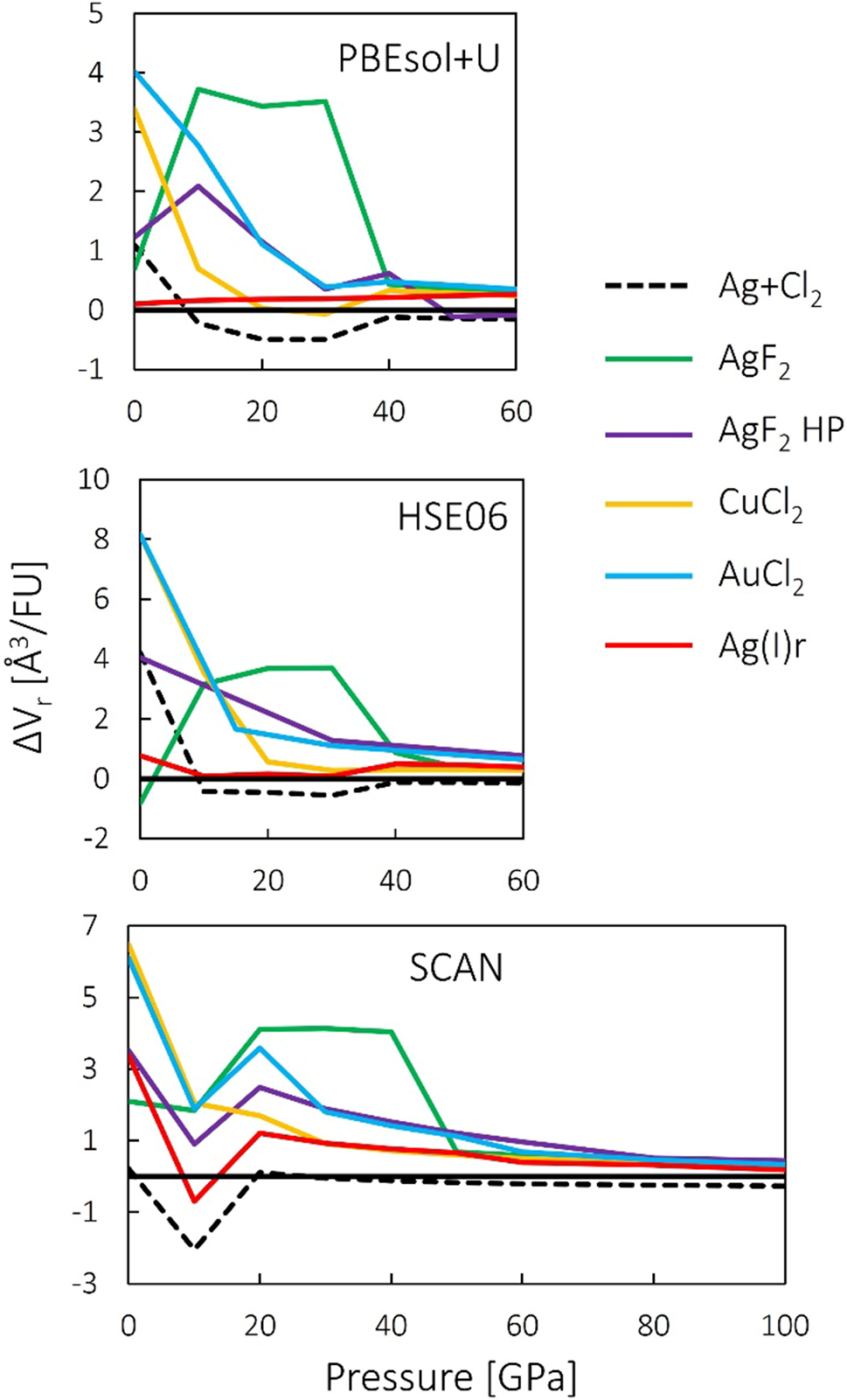
Volume of selected studied polymorphs of AgCl_2_ relative to AgCl + ½Cl_2_. Top panel—PBEsol + U, middle panel—HSE06 (hybrid functional), bottom panel—SCAN (meta-GGA functional). *FU* formula unit.

The initial set of calculations was performed within the PBEsol + U approach, as the least computationally demanding. It was found that the Ag(I)h type collapses upon compression to 10 GPa into the Ag(I)r structure and it was not considered any further in the analogous HSE06 and SCAN calculations. Similarly, MnO_2_ type was also neglected past the PBEsol + U approach, as it was found to be the least stable in terms of ΔH_r_ among the studied types. Therefore, these two polymorphs are not taken into account in Figs. 1, 2 and 3.

Figure 3 compares relative volume (ΔV_r_) of the studied polymorphs with respect to AgCl + ½Cl_2_ mixture. Since the *pV* term becomes a considerable contribution to enthalpy at elevated pressures, the fact that most of the candidates for AgCl_2_ considered here have a positive ΔV_r_ can be seen as an important factor leading to their relative instability. However, it should be noted that only HSE06 accurately reproduces ambient-pressure volumes of AgCl and Cl_2_. PBEsol + U underestimates the volume of AgCl and Cl_2_, while SCAN—that of Cl_2_. Therefore, ΔV_r_ values at 0 GPa in Fig. 3, and by extension—initial compressibilities—should be taken with a grain of salt. A noticeable dip at 10 GPa within the SCAN approach is likely a manifestation of this. The computationally demanding HSE06 results are likely to be the most correct.

Some of the features in Figs. 2 and 3, such as abrupt changes of relative energies or volumes, and apparent convergence of plots corresponding to different polymorphs are indicative of structural transitions. The nature and implication of those transition will be discussed in the next section.

### Pressure-induced structural transitions

As an introduction to analysis of structural transitions of AgCl_2_ polymorphs, let us first discuss the Ag(I)r solution. As mentioned before, this structure is made up of subunits of rocksalt-type AgCl and of Cl_2_ molecules. Within the studied pressure range, it undergoes structural rearrangements, which can be approximated as a sequence of deformations of the AgCl double layers, leading from a fundamentally NaCl-like coordination patterns to CsCl-like patterns, with increasing coordination number of Ag atoms. Importantly, it should be stressed again that this structure emerged as one of the lowest-energy solutions in an evolutionary algorithm structural search reported in the previous contribution^6^. It should not be treated as a viable candidate for the structure of AgCl_2_, but rather as a manifestation of the proclivity of the studied system towards charge transfer and phase separation. The case for instability of Ag^II^Cl_2_ towards these processes is further strengthened by the fact that the Ag(I)r polymorph remains the most stable (with respect to ΔH_r_) throughout the studied pressure range in both PBEsol + U and HSE06 approaches. A noticeable drop in ΔH_r_ between 50 and 60 GPa for this polymorph in the SCAN approach was the reason for extending the studied pressure range to 100 GPa in this case. However, this drop is a result for NaCl-CsCl-like transition in the AgCl subphase, which is more abrupt than in analogous PBEsol + U and HSE06 calculations. No further phase transitions for Ag(I)r polymorph are observed up to 100 GPa.

**Figure 4:**
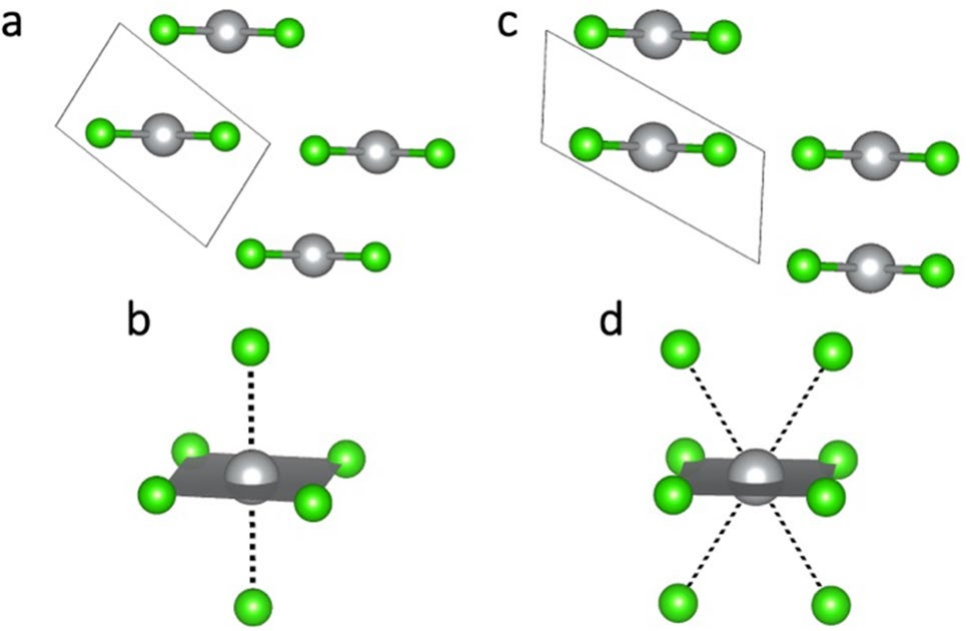
Structural changes in CuCl_2_-polymorph. **(a,c)** View along the direction of chain propagation at 10 and 20 GPa, respectively. **(b,d)** View of local coordination of Ag at 10 and 20 GPa, respectively. Ag—grey, Cl—green.

The CuCl_2_-type polymorph emerges as the most structurally robust in this study, as it does not undergo any collapse or drastic deformation in the studied pressure range, maintaining a relatively low ΔV_r_ in all three methods. The only change to the structure of this polymorph occurs in terms of arrangement of chains relative to one another. Up to 10 GPa (in all three computational approaches), the chains are positioned as in Fig. 4a, resulting in octahedral coordination of Ag atoms (Fig. 4b). The octahedra are elongated by 29%, 29% and 25%, according to PBEsol, HSE06 and SCAN, respectively. By account of all three methods, the arrangement changes between 10 and 20 GPa, leading to a 4 + 4 coordination of Ag atoms, with the 4 inter-chain contacts longer by 23 to 33%, depending on the method (Fig. 4d). This is achieved in different ways: in PBEsol + U and HSE06 results, the chains move relative to one another in a direction perpendicular to direction of propagation (Fig. 4c). In SCAN approach, this is achieved through a change of one of the unit cell angles, which results in sliding the chains relative to each other. The resulting 4 + 4 coordination is the same in all cases (Fig. 4d), but the longer Cl contacts are aligned parallel (in SCAN) or perpendicular (in PBEsol + U and HSE06) to direction of propagation. Further compression to 30 GPa transforms the structure into that seen at 20 GPa in PBEsol + U and HSE06 pictures. All of these transitions can be seen as a means to achieve a more efficient packing of chains (as evidenced by increasing coordination number of Ag). Changes in local coordination of Ag atoms in this polymorph are plotted in the top panel of Fig. 6.

Another result of these rearrangements is a reduction of Cl…Cl distances between neighboring chains. These distances drop from ca. 3.8 to 2.7 Å between 0 and 60 GPa in HSE06, compared to 3.2 to 2.5 Å in PBEsol + U and 3.5 to 2.8 Å in SCAN. Recall that only HSE06 correctly reproduced the ambient pressure (low-temperature) volume of solid molecular Cl_2_ at 0 GPa, so these results testify to the superiority of HSE06 in describing weak interactions compared to the other two methods utilized here (and free from the explicit van der Waals terms). Importantly, all of these distances are larger than the Cl-Cl bond in solid molecular Cl_2_, which remains at ca. 2.00 Å and contracts very little (less than 0.05 Å) with compression in results from all three methods. Additionally, this polymorph was further optimized at 100 GPa with PBEsol + U and SCAN methods, and does not undergo any structural modifications in that pressure range.

**Figure 5:**
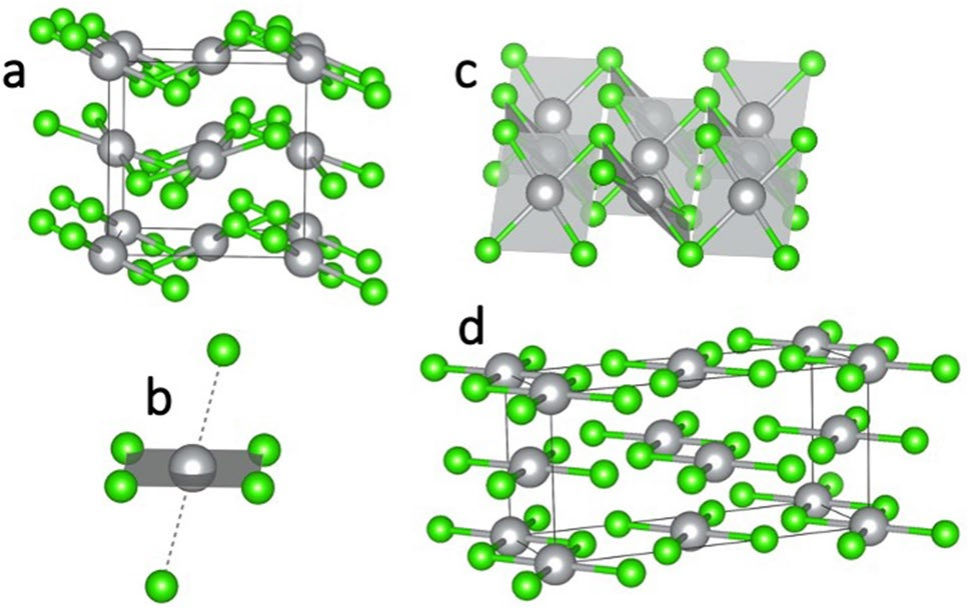
Structural features of AgF_2_-type polymorph: **(a)** ambient-pressure structure; **(b)** local coordination of Ag in the ambient-pressure structure; **(c)** one layer at 40 GPa in HSE06 approach—note that axial Cl atoms are now within the same layer, a pair of those contacts is marked with red dashed line; **(d)** structure resulting from phase transition between 40 and 50 GPa (30 and 40 GPa in PBEsol + U approach). Note the CuCl_2_-like chains. Ag—grey, Cl—green.

**Figure 6:**
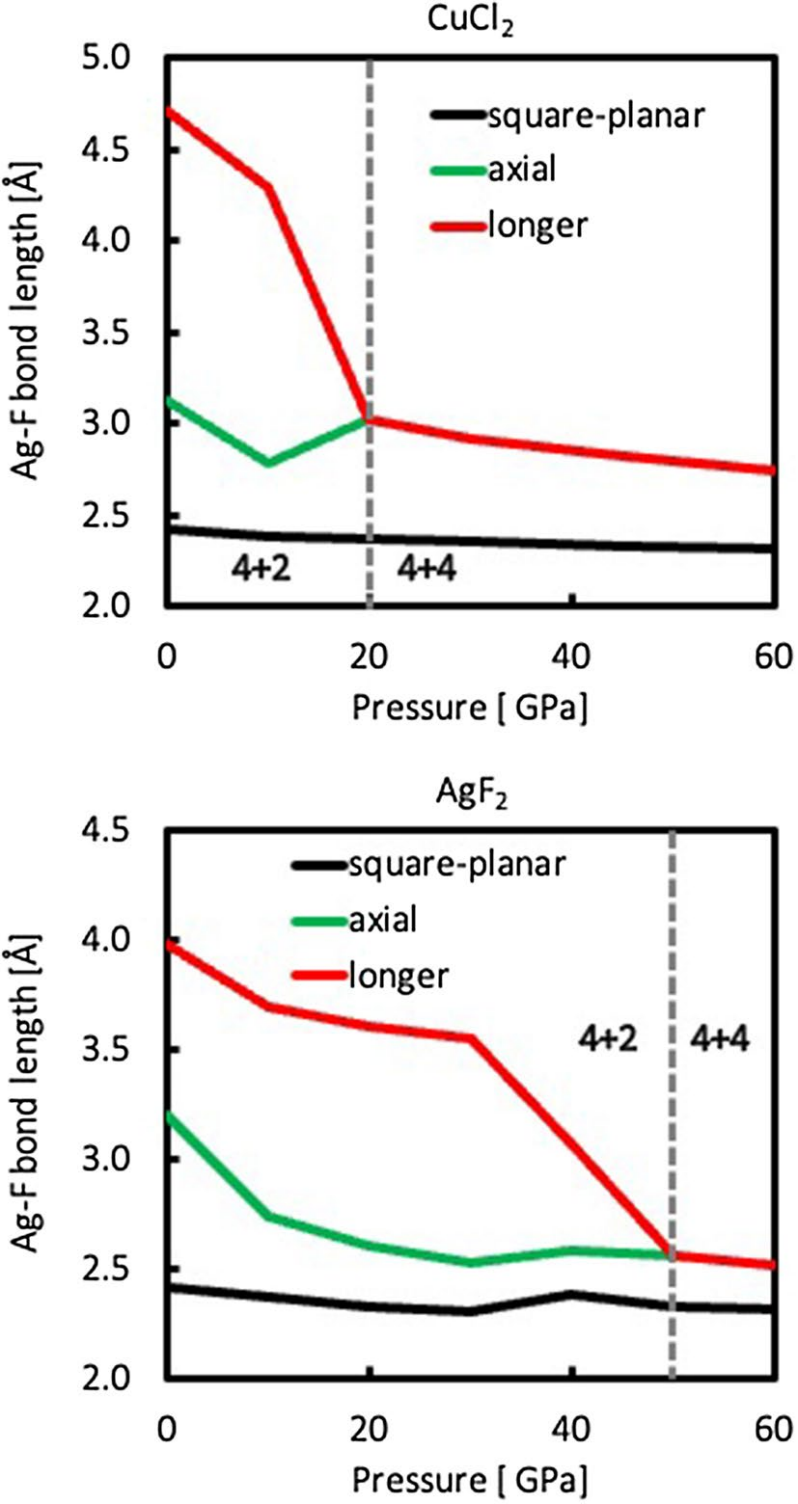
Pressure dependence of Ag-F distances in CuCl_2_-type and AgF_2_-type polymorphs in the HSE06 picture.

AgF_2_-type polymorph undergoes substantial structural rearrangements with increasing pressure. At 0 GPa, it adopts a layered structure of *Pbca* space group (Fig. 5a), in which every Ag atom forms 4 in-layer bonds with Cl atoms, with two additional Cl atoms from adjacent layers together constituting a distorted octahedral coordination (Fig. 5b). The axial Cl contacts are noticeably longer (by 23%, 33% and 27% in PBEsol + U, HSE06 and SCAN, respectively) than equatorial ones. The same phenomenon is observed in AgF_2_ and in general, elongated octahedral coordination is a well-documented phenomenon in silver(II) fluorides (which includes ternary compounds)^10^. This is usually attributed to Jahn–Teller effect, whereby a vibronic instability leads to elongation or contraction of bonds along one of the three axes of octahedron, which lowers the overall electronic energy. Upon compression, this elongation is reduced in AgCl_2_ down to 1%–2% at 30 GPa in both PBEsol + U and SCAN results. The structure at this point is reminiscent of high-pressure structure of PdF_2_^11^, which—like AgF_2_—can be described as a distorted fluorite, but lacks Jahn–Teller elongation of [PdF_6_] octahedra and is cubic (*Pa*-3). Further increase of pressure (40 GPa in PBEsol + U and 50 GPa in SCAN) leads to a structural transition into a polymorph consisting of 1D chains similar to those found in CuCl_2_-type polymorph (Fig. 5d). The symmetry of this collapsed chain polymorph corresponds to *Pnma* space group. In the HSE06 picture, the contraction is less pronounced—down to 10% at 30 GPa, and the transition to the chain polymorph occurs via a different layered structure observed at 40 GPa, where the layers become more corrugated and more separated from each other (Fig. 5c). Ag atoms retain an approximately octahedral coordination, but the axial Cl contacts are now within the same layer as [AgCl_4_] squares. The elongation of octahedra due to Jahn–Teller effect is still noticeable (9%). Further compression to 50 GPa leads to a collapse to chains as in the other two methods. Changes in local coordination of Ag atoms in this polymorph are plotted in the bottom panel of Fig. 6.

The transitions described above can be seen as an abrupt drop in relative enthalpy in Fig. 2. In the previous contribution discussing relative stability of AgCl_2_ candidate structures, AgF_2_-type emerged as the most likely candidate at higher pressures due to its comparatively low molar volume among the considered structures^6^. However, it appears that compression of AgF_2_-type produces a lot of strain in the structure, as evidenced by a strong increase of ΔH_r_ (Fig. 2), which is released through the aforementioned transition.

**Figure 7:**
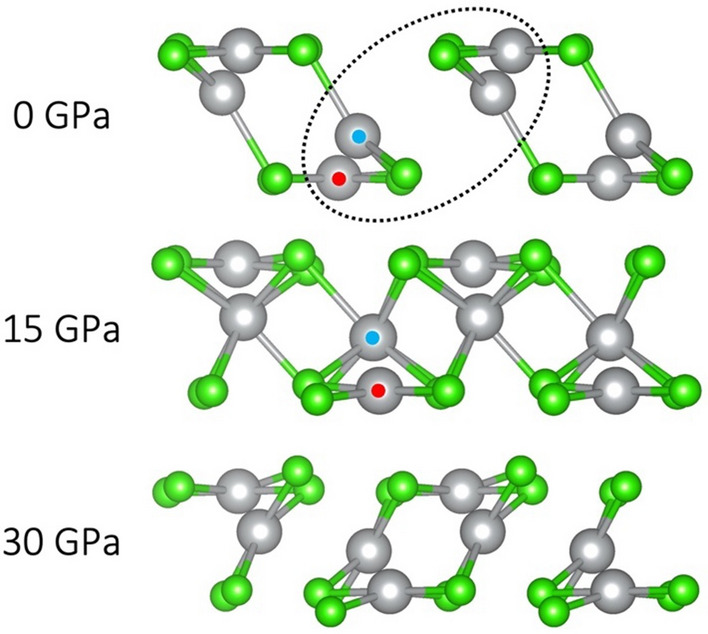
Structural transition of AuCl_2_-type polymorph in the HSE06 picture. Nanotubes are viewed along the axis of propagation. Blue and red circles indicate Ag(I) and Ag(III) species, respectively.

Arguably the most interesting pattern of pressure-induced transitions can be observed in the AuCl_2_-type nanotubular polymorph. The differences in outcomes of compression between the three computational approaches are the most pronounced for this system, although upon closer look we can identify their fundamental similarity. Recall that in the HSE06 approach, AuCl_2_-type is mixed-valent: Ag(I) species are connected to 3 Cl atoms in an approximately flat triangular pattern, while the Ag(III) sites appear as [AgCl_4_] square units, which are analogous to those in AgF_3_^12^. At 0 GPa, the triangular contacts average 2.53 Å, while the bonds in square units are 2.29 Å, which is even shorter than for Ag(II) in [AgCl_4_] square in CuCl_2_-type and AgF_2_-type polymorphs at the same pressure. This supports the assignment of the sites as Ag(I) and as Ag(III), respectively. Compression to 15 GPa induces a change in local coordination of the Ag(I) species, which picks up 4 Cl atoms along the axis perpendicular to the plane of the former triangle, resulting in a 4 + 3 coordination, with an average bond length of 2.61 Å. Meanwhile, the Ag(III) subunit retains a square coordination with a shorter average bond length of 2.27 Å. Further compression to 30 GPa leads to a rearrangement of nanotubes, which are now formed by a different combination of Ag and Cl atoms; importantly, all Ag atoms are coordinated by 4 Cl atoms in an approximately square-planar manner, with an average for the formerly Ag(III) sites at 2.33 Å and the formerly Ag(I) ones—2.38 Å. This convergence of the two sites in terms of local coordination points to a comproportionation process, whereby all Ag sites are now nominally Ag(II) species. The transition described above is shown in Fig. 7.

**Figure 8:**
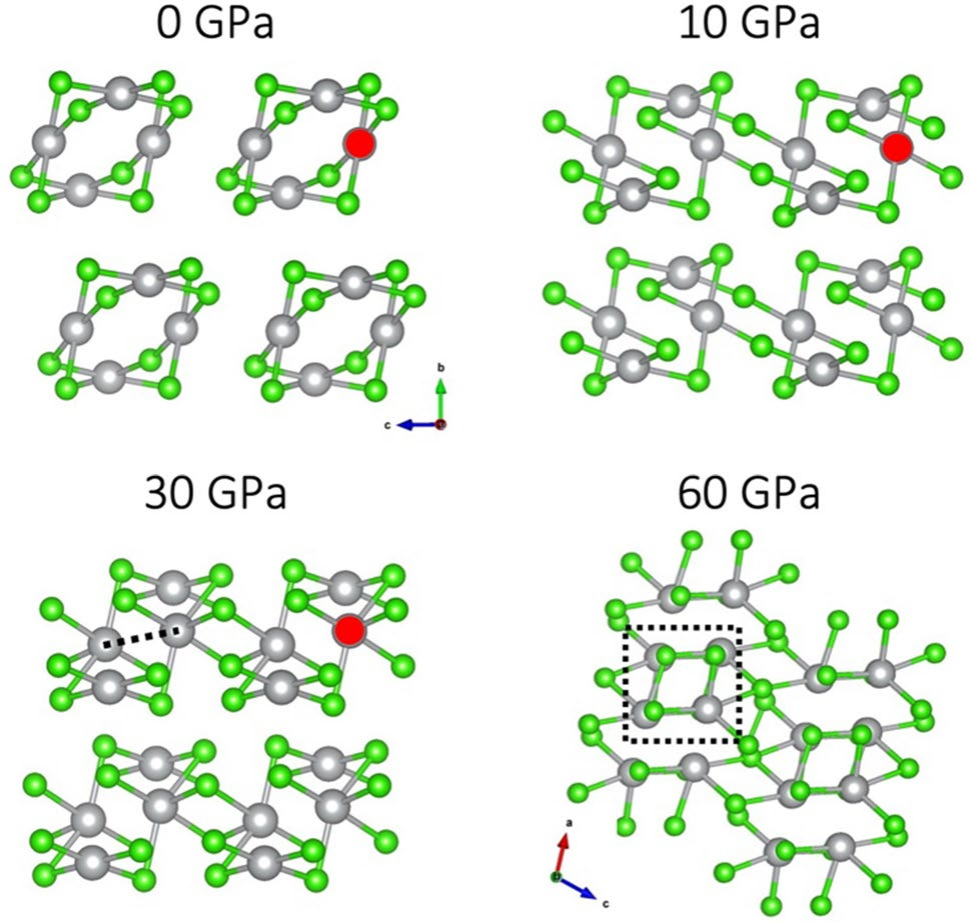
Structural transition of AuCl_2_-type polymorph in the SCAN picture. Nanotubes are viewed along the axis of propagation from 0 to 30 GPa. Note a different projection at 60 GPa, along the newly formed [AgCl] square nanowires. Red circles mark the Ag atom which experiences the pronounced changes in local coordination in the 0–30 GPa range.

A very different scenario is observed in the SCAN picture (Fig. 8). At 0 GPa, the two Ag sites are already equivalent, but upon compression to 10 GPa, the local square-planar coordination of one of them is rotated by 90 degrees i.e., two of its four nearest Cl neighbors are substituted for another two, which leads to connections between nanotubes in the ***c*** direction. Further compression to 30 GPa results in an inward contraction of individual nanotubes. The local coordination of the Ag sites marked with red circle changes to more uniformly octahedral—a fifth Cl atom is picked up from a neighboring nanotube along the axis perpendicular to the [AgCl_4_] plane. The average length of the five Ag–Cl bonds for this Ag site is 2.51 Å (2.45–2.55 Å). The sixth nearest neighbor, on the opposite side of the former [AgCl_4_] is actually another Ag atom, located at a distance of 2.70 Å. (This connection is marked with a dashed line in Fig. 8.) Importantly, this new Ag…Ag contact is consistently shorter than the Ag–Ag distance in metallic silver at corresponding pressures (from SCAN calculations): 2.70 Å vs. 2.72 Å (30 GPa), 2.64 Å vs. 2.69 Å (40 GPa) and 2.60 Å vs. 2.67 Å (50 GPa). This leads us to infer a formation of a weak Ag–Ag interaction in this polymorph, which is a very interesting finding and reminiscent of a recent work, where a silver subchloride Ag_8_Cl_6_ was predicted, featuring [Ag_6_] subunits within its structure^13^. Further compression above 50 GPa leads to a substantial structural rearrangement: AgCl-like chains with square cross-section are formed, interspersed with Cl_2_ molecules.

**Figure 9:**
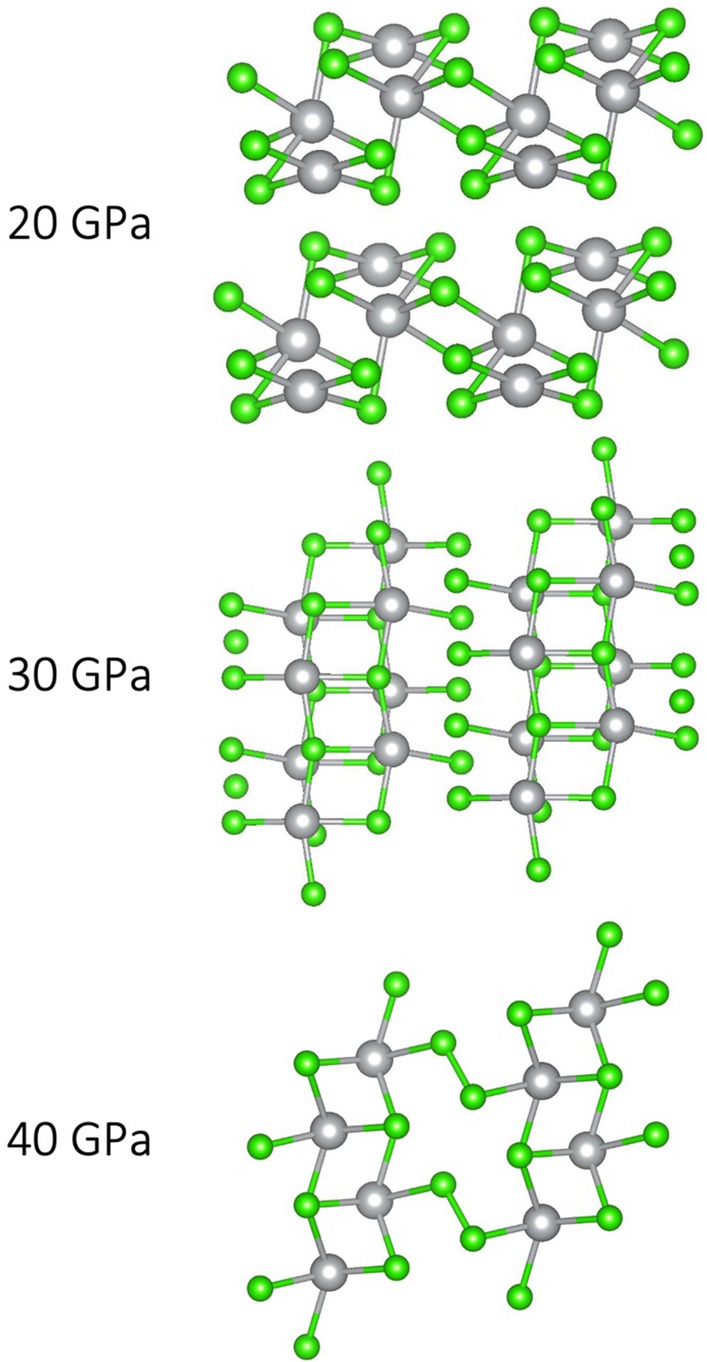
Structural transitions of AuCl_2_-type polymorph in the PBEsol + U picture.

A similar picture emerges from the PBEsol + U approach (Fig. 9). As in SCAN results, compression to 10 GPa leads to a rearrangement of local coordination of half of Ag sites. The subsequent contraction of nanotubes and formation of short Ag…Ag contacts is observed at a lower pressure of 20 GPa (compared to 30 GPa in SCAN). Upon further pressure increase to 30 GPa, a structure similar to Ag(I)r polymorph is formed, consisting of double layers of rocksalt-like AgCl interspersed with Cl atoms. The difference is that here, no discernible Cl–Cl molecules are formed—the distance between Cl atoms lying between AgCl layers is ca. 2.3 Å, compared to ca. 2.0 Å in solid molecular Cl_2_ and in Ag(I)r polymorph. This is likely an artificial result, which will be further discussed in the “Electronic structure” section. Finally, compression to 40 GPa leads to rearrangement within AgCl layers, which increases the coordination number of Ag from 6 to 7 and the coordination environment resembles that in CsCl. Cl_2_ molecules between the AgCl layers, characteristic of Ag(I)r polymorph, can also be discerned. Indeed, as can be seen in Fig. 2, this solution also converges with Ag(I)r polymorph in terms of ΔH_r_.

As in the case of Ag(I)r solutions, formation of separate domains of AgCl and Cl_2_ during structural transitions of AuCl_2_-type in PBEsol + U and SCAN picture, can be interpreted as another manifestation of the system’s tendency for AgCl + ½Cl_2_ phase separation, rather than as viable structural candidates. However, it should be pointed out that sodium and potassium chlorides with exotic stoichiometries as e.g. NaCl or Na_3_Cl, have been predicted in the past^14,15^.

Nanotubular AgF_2_-HP-type polymorph undergoes a structural collapse above 30 GPa in PBEsol + U results and above 60 GPa in SCAN results. The final structures do not resemble any of those discussed above; rather, they feature domains of connections between Ag and Cl atoms which do form any extended and discernible pattern, and are instead interspersed with Cl_2_ molecules. Since those solutions are consistently very high in relative enthalpy (ΔH_r_), they will not be further analyzed here. (However, an example of the resulting structures can be found in Supplementary Information).

### Dynamical stability of candidate AgCl_2_ structures

**Figure 10:**
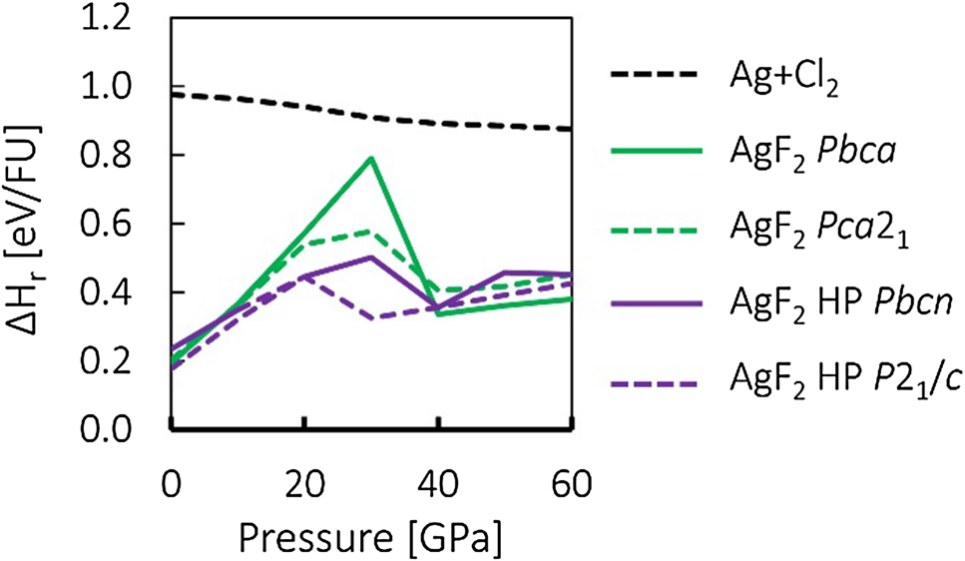
Comparison of ΔH_r_ pressure dependence for original (solid line) and phonon-relaxed (dashed line) structures of AgF_2_ and HP-AgF_2_ polymorphs of AgCl_2_.

In addition to the analysis of relative enthalpy of hypothetical polymorphs of AgCl_2_, we also investigated stability of their crystal structures by calculating (at the PBEsol + U level) phonon frequencies in Γ point of the first Brillouin zone, in the same unit cells as presented above. We found that the Ag(I)r and AuCl_2_ types exhibit no imaginary phonon frequencies at Γ point in the studied range from 0 to 60 GPa. CuCl_2_-type polymorph exhibits two imaginary frequencies only at 0 GPa, which, when followed along their normal coordinates and relaxed, lead to the same structure as the original solution at 0 GPa (within 0.01 meV/FU in terms of total energy), so these may be considered as artifacts. We have, however, found structural instabilities in AgF_2_-type and nanotubular HP-AgF_2_-type, which we discuss below. Figure 10 compares pressure dependence of ΔH_r_ for the original (high symmetry) and phonon-relaxed (lower symmetry) structures in the two cases mentioned.

**Figure 11:**
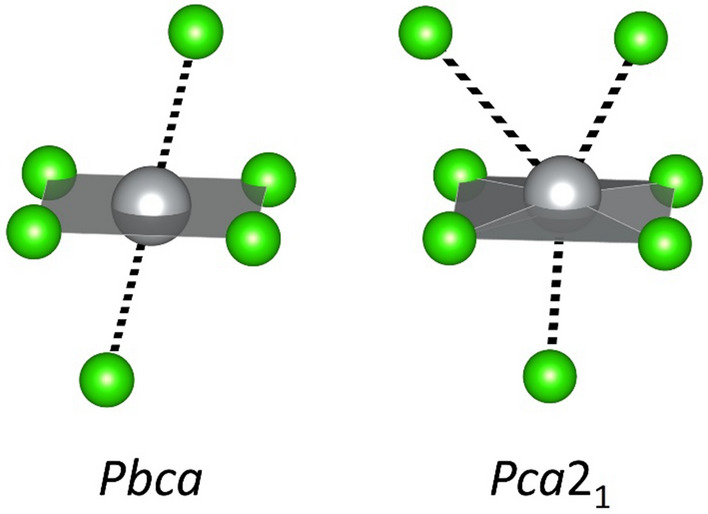
Comparison of local coordination of original and phonon-relaxed structures of AgF_2_-type at 10 GPa.

The layered AgF_2_-type polymorph is dynamically stable at 0 GPa, but at 10 GPa, an imaginary mode of B_1u_ symmetry emerges, which leads to a structure analogous to the previously reported non-centrosymmetric (*Pca*2_1_), moderate-pressure polymorph of AgF_2_^4^. This solution retains the layered arrangement, but the Ag atoms diverge from their position within the plane formed by surrounding Cl atoms (Fig. 11). The consequence of this transition is that the local coordination of Ag atoms changes from 4 + 2 (deformed octahedron) to 4 + 3 (Fig. 11), which likely minimizes repulsion between one of the lobes of the filled d(z^2^) orbitals and ligands. We proceeded to optimize this new solution in the same pressure range as the original AgF_2_ (0–60 GPa, every 10 GPa). At 0 GPa, the original *Pbca* remains more stable (by ca. 0.01 eV/FU in terms of ΔH_r_). At 10 GPa, it becomes only slightly favored (by 0.004 eV/FU in terms of ΔH_r_), and more favored at higher pressure up to 30 GPa. The *Pca*2_1_ retains dynamic stability (no imaginary phonons in Γ) until 30 GPa. At higher pressure—like the original *Pbca* solution—it collapses into a 1D structure, which is higher in energy than the previously discussed *Pnma* structure resulting from the collapse of *Pbca* (Fig. 10). The new collapsed structure features chains of AgCl interspersed with chains of Cl atoms along the *c* direction (the structure can be found in ESI).

**Figure 12:**
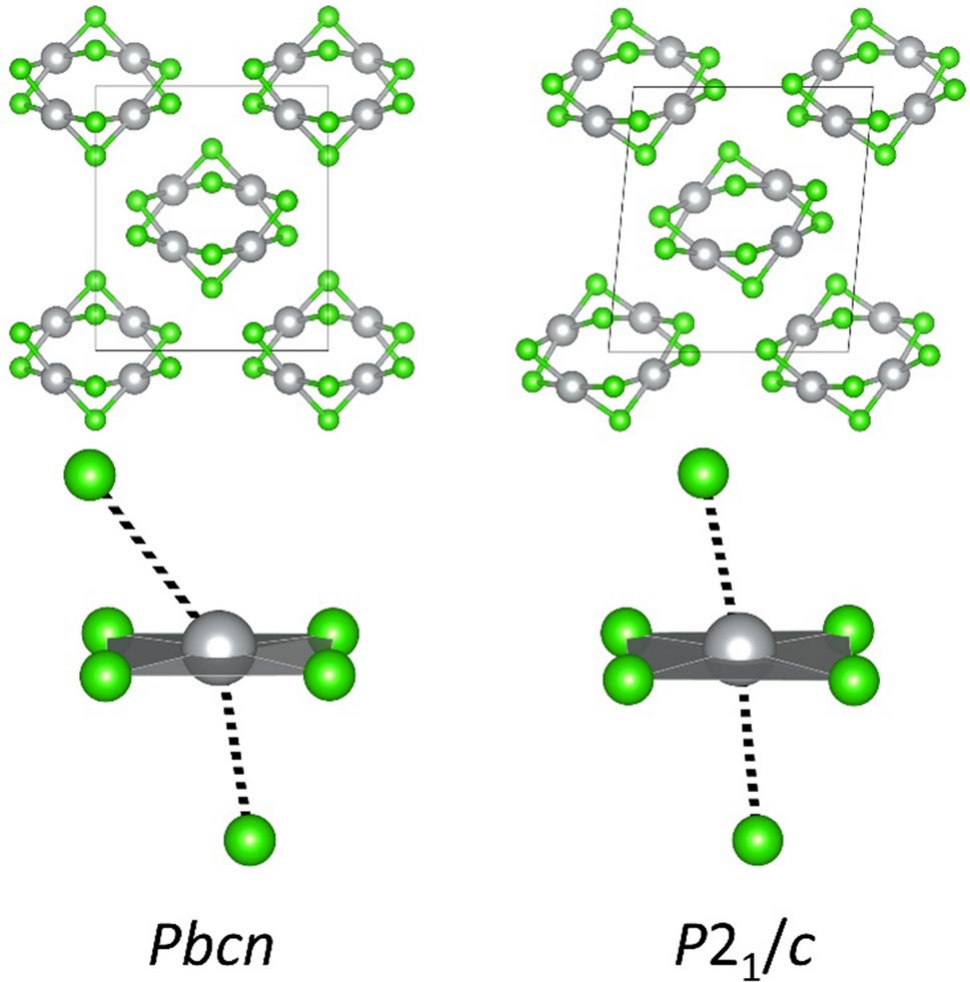
Comparison of structures and local coordination Ag atoms in original and phonon-relaxed structures of HP-AgF_2_-type at 0 GPa.

Similarly, nanotubular HP-AgF_2_ type exhibits an imaginary mode of B_1g_ symmetry already at 0 GPa. Following a normal coordinate of that mode and relaxing the structure, we obtain a lower-symmetry nanotubular structure (*P*2_1_/*c* space group), which is more stable that the original in terms of ΔH_r_ by 0.06 eV/FU already at 0 GPa. Likewise, we re-optimized this new phonon-relaxed solution in the 0–60 GPa range. The *P*2_1_/*c* solution remains more stable that the original *Pbcn* in terms of ΔH_r_ up until its own collapse above 20 GPa (a drop between 20 and 30 GPa can be seen for the purple dashed line in Fig. 10). It also remains dynamically stable (lack of imaginary phonons in Γ) within that range. Figure 12 presents the change in local coordination experienced by Ag atoms upon aforementioned relaxation at 0 GPa.

Importantly, for the Ag(I)r-type as well as CuCl_2_-type polymorphs at 0 GPa (i.e. the two most stable ones in terms of ΔH_r_) we also calculated full phonon dispersion curves at the PBEsol + U level (in 2 × 2 × 2 and 1 × 2 × 2 supercells, respectively), which are shown in Supplementary Information. The results point to lack of imaginary phonons, which indicates metastability of these proposed structures. This leaves chance for the conceivable existence of these phases if prepared by some mildly exothermic reaction.

### Electronic properties

As inferred from the previous paper^6^, antiferromagnetic superexchange in Ag^II^Cl_2_ can, in principle, be expected to be strong, since Ag–Cl bonding in this hypothetical compound would likely be more covalent in nature than in its AgF_2_ counterpart. Of course, AgCl—the only currently known binary combination of silver and chlorine—is an ionic solid, as is AgF. However, previous studies of AgF_2_ demonstrated a covalent character of Ag–F bonding in that compound, evidenced by X-ray photoelectron spectroscopy^16^ and by optical spectra^17^. In the former study, covalency increased in the sequence AgF → AgF_2_ → AgF_3_. Therefore, it is reasonable to expect a similar trend in AgCl_x_ compounds. On the other hand, Cl^–^ anions are larger and more diffuse and are therefore softer Lewis bases than F^–^ anions, which makes them more vulnerable to the strongly oxidizing properties of Ag(II) cations. In addition, increasing pressure and the consequent reduction of interatomic distances increases orbital overlap, leading to broadening of electronic bands, which ultimately results in metallization of most known compounds (both ionic and covalent)^7^.

Electronic properties of the studied AgCl_2_ candidate polymorphs were scrutinized in terms of (a) magnetic moments on Ag atoms and (b) fundamental band gap at the Fermi level in electronic density of states (eDOS) graphs. As it turns out, changes in the two parameters are strongly correlated in that the pressure at which the band gap closes coincides with disappearance of magnetic moment on Ag atoms.

**Figure 13:**
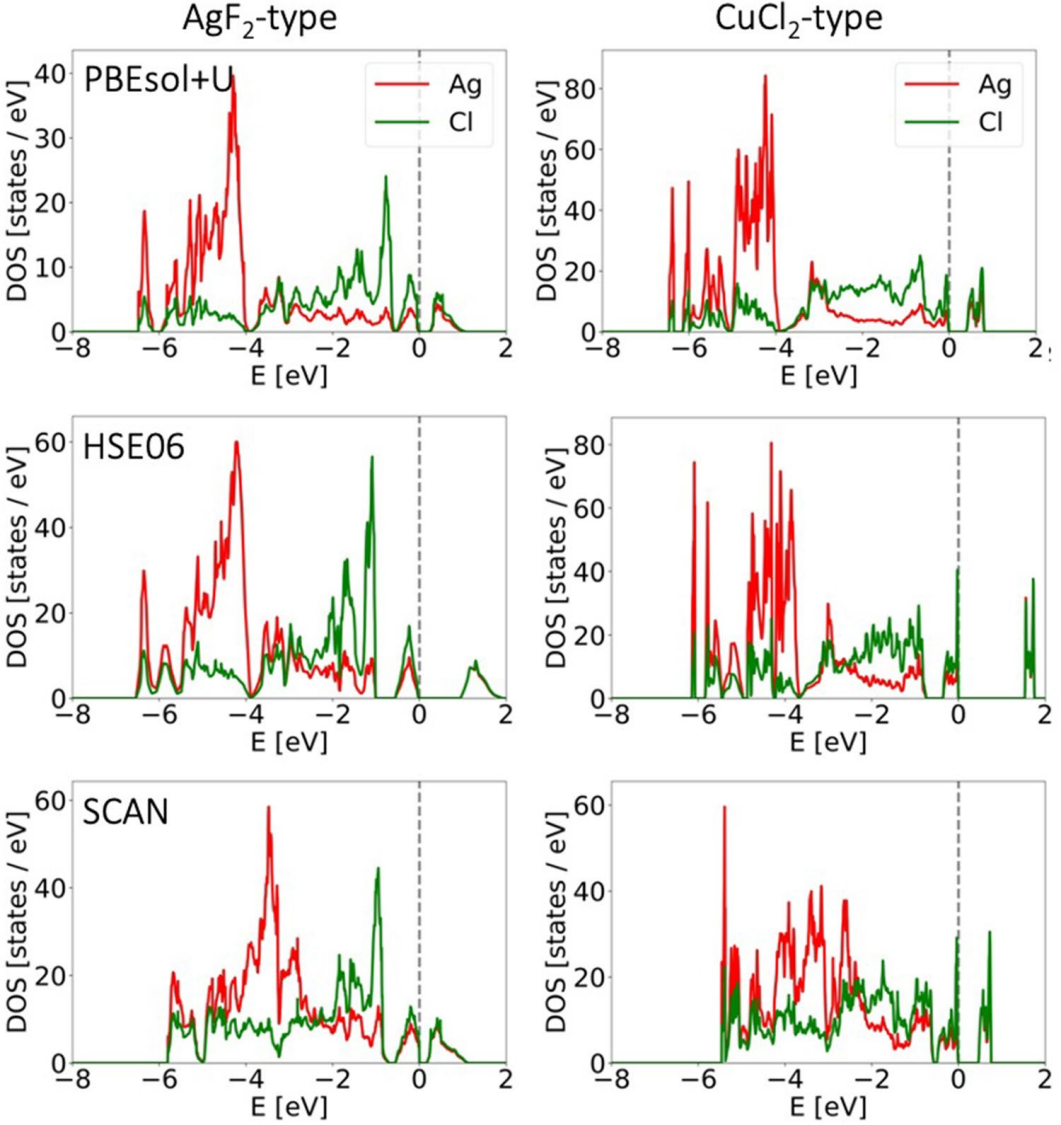
Comparison of eDOS plots for AgF_2_-type and CuCl_2_-type between different computational methods at 0 GPa.

An example of eDOS plots—for AgF_2_-type and CuCl_2_-type—at 0 GPa and comparison between the three computational methods is presented in Fig. 10. One noticeable feature is the composition of conduction band. In principle, AgCl_2_, just like the known AgF_2_, is expected to be a charge-transfer insulator, where the band gap arises between filled nonmetal states and empty metal states (upper Hubbard band, UHB)^18^. While this is certainly the case in AgCl_2_, we can see a substantial admixing of Cl states to the conduction band, with an almost perfect overlap and approximately equal contributions from Ag and Cl states. This indicates a strong covalence of the Ag–Cl bonds that is comparable or indeed even stronger than in AgF_2_^16^. It should also be pointed out that “insulator” in this case refers to a non-zero band gap resulting from electronic correlation as per the aforementioned Zaanen-Sawatzky-Allen model^18^. Clearly, with a band gap in the range 0.2–1.5 eV (depending on the method) (Fig. 13), the two AgCl_2_ polymorphs in question can be more accurately described as semiconductors.

Table 1 compares metallization pressure for selected polymorphs and between three methods. Metallization pressure is defined here as the lowest pressure point at which a solution exhibits null magnetic moments and null band gap. It can be seen that this value is the highest in HSE06 results. This coincides with the observation that the band gap is the largest for AgF_2_-type and CuCl_2_-type polymorphs in the HSE06 picture (Fig. 13). The differences between methods likely stem from the inclusion of exchange correlation in the hybrid-DFT-type functional, while in GGA-type PBEsol + U approach, electronic correlation, which is crucial for modelling open-subshell systems such as Ag(II) compounds, is only taken into account through *U* and *J* parameters (mentioned in “Methods” section). HSE06 results can likely be considered the most accurate, since—in general—hybrid functionals like HSE06 are very well suited for predictions of band gaps in solids (compared to GGA and even meta-GGA)^19^.

**Table 1:** Pressure of metallization for different polymorphs of AgCl_2_.

	PBEsol + U	HSE06	SCAN
AgF_2_-type	20 GPa	> 60 GPa^a^	10 GPa
CuCl_2_-type	10 GPa	45 GPa	10 GPa
AgF_2_ HP-type	0 GPa	60 GPa	10 GPa
AuCl_2_-type^b^	0 GPa	15 GPa^c^	10 GPa

^a^Although transformed into chains similar to CuCl_2_-type, the polymorph retains residual magnetic moment and non-zero band gap at the maximal studied pressure of 60 GPa. Magnetic coupling is FM intra-chain and AFM inter-chain.

^b^Reopening of band gap occurs at higher pressures—see text.

^c^This polymorph is mixed-valent in HSE06 picture and is non-magnetic at 0 GPa.

The phase-separated Ag(I)r polymorph remains insulating within the studied pressure range and by account of all three methods. Structural transitions into phase-separated solutions observed for AuCl_2_-type polymorph, which were discussed in the previous section, are also associated with reopening of the band gap. That is because constituent parts of these solutions—sublattices made up of ionic AgCl and of molecular Cl_2_—are insulators. The fact that the solution for AuCl_2_-type polymorph at 30 GPa in PBEsol + U picture retains metallic character after the afomentioned transition stems from the presence of dangling, unpaired Cl atoms on the outside of AgCl layers. Such arrangement should be unstable towards a Peierls distortion and formation of Cl_2_ molecules, which is indeed what happens upon further compression to 40 GPa, and in SCAN picture. This testifies to the relatively poor suitability of PBEsol + U for describing electronic correlation.

## Discussion

The Ag + Cl_2_ phase diagram, which was studied here, consists of substances featuring a broad spectrum of chemical bonding types: metallic Ag, molecular Cl_2_, ionic AgCl, as well as a variety of more or less covalently bonded polymorphs of AgCl_2_, all of which have their own specific challenges when it comes to accurate theoretical description. Since AgCl_2_ has not yet been observed experimentally, it is difficult to judge which of the three methods—GGA DFT (PBEsol + U), hybrid-DFT (HSE06) and meta-GGA DFT (SCAN)—is the most trustworthy for the assessment of stability of AgCl_2_ at higher pressures—in particular, whether the region of stability seen in SCAN picture can be considered a reasonable finding. Our conclusions can to some extent be based on the results that these methods have provided when previously applied to other similar systems. To the best of our knowledge, a comparative computational study of an extended solid system comprised of a transition metal halide—like this one—has not been previously reported in literature. However, computations with hybrid functionals have been shown to provide excellent agreement with experimental data for e.g. ionic halides, where they are able to accurately reproduce lattice constants and bulk moduli of these solids^20^. In general, hybrid functionals appear to have the potential to be the most accurate among DFT methods, superior to both GGA and meta-GGA^21^. Additionally, in the case of our system, HSE06 functional is the only one of the three methods which correctly reproduces the volume and intermolecular distances in solid Cl_2_, which are determined by weak interactions between molecules.

Having said that, it is important to note that the overall picture which emerges from data presented here is remarkably consistent across the three computational methods, differing mostly in terms of pressure at which structural and electronic transitions are observed. Transitions of CuCl_2_ and AgF_2_ types reveal the tendency of the system to avoid repulsion between axial Cl atoms and filled d(z^2^) orbital of Ag atoms—through relative displacement of chains in the former and through transition from a layered 2D structure into 1D chains in the latter. A similar tendency is seen in pressure-induced phase transitions of AgF_2_, where the high-pressure nanotubular structure can be viewed as a means both to maximize coordination number and to minimize the repulsion from the Ag d(z^2^) lone pair^4^. The transitions of AuCl_2_-type and the relative stability of Ag(I)r polymorph firmly indicate that Ag^II^Cl_2_ is unstable towards charge transfer and phase separation into AgCl and Cl_2_. Fundamentally, phase transitions observed in the studied polymorphs unfold upon pressure-induced decrease in distance between 1D or 2D structural constituents (chains, nanotubes, layers), which entails overcoming weak repulsive interactions between them. Among the three methods utilized here, the HSE06 functional, for reasons summarized above, is most likely best suited for description of those interactions.

Results reported in this work shed some light on properties and prospects of synthesis of the hypothetical AgCl_2_. Most importantly, our study shows that high pressure does not stabilize the candidate polymorphs derived from the previous study—in particular, we see that the prediction of stability of AgF_2_-type polymorph, which was based on a reasonable extrapolation from ambient-pressure data, nevertheless turned out not to be false^6^.

Of course, this work does not exhaust the list of possible candidates for the structure of AgCl_2_. Although the studied candidates generally retain a positive enthalpy with respect to decomposition into AgCl + ½Cl_2_, it is worth noting that the CuCl_2_-type chain structure does not collapse into phase-separated polymorph even at 100 GPa in PBEsol + U and SCAN results. Analogous HSE06 calculations at 100 GPa were not performed, but based on the observation made here that transition pressures are reliably the highest within HSE06 approach, it is reasonable to predict that such collapse would not be seen in HSE06 picture at 100 GPa, either. The apparent lack of such transition pathway could mean that, in principle, obtaining it as a metastable phase could be possible if e.g., elevated temperatures are used together with moderate pressures. Our previous results^6^, as well as earlier predictions by other authors^22,23^, all consistently suggest that Ag^II^Cl_2_, if ever obtained, would likely be metastable.

Although the current study was focused on AgCl_2_ stoichiometry only, the results of this study permit us to extrapolate observed trends towards the AgCl_3_. The tendency of AgCl_2_ stoichiometry to undergo decomposition to (AgCl)(Cl_2_)_½_ may suggest that even at larger Cl contents, i.e. for AgCl_3_ stoichiometry, one will observe phase separation to (AgCl)(Cl_2_), or Ag^+^(Cl_3_^–^)^24^. A similar result is indicated by theoretical study for isolated AgCl_3_ molecules in the gas phase^25^.

## Methods

Calculations were carried out using VASP software^26–30^. Overall, three different computational methods were utilized (underlined are the names by which they are referred to throughout this work):

I. PBEsol + U approach: GGA-type Perdew–Burke–Ernzerhof functional adapted for solids (PBEsol)^31^ was used, additionally taking into account Coulombic interactions between d electrons through U and J parameters^32^ explicitly set to 5 eV and 1 eV, respectively, and with correction for van der Waals interactions^33^. Plane-wave cutoff energy was set to 800 eV. k-space sampling of ca. 2π × 0.04 Å^−1^ was used for optimization and a denser k-spacing of ca. 2π × 0.03 Å^−1^ was used for electronic density of states (eDOS) calculations. On an example structure of CuCl_2_-type, this corresponds to 2 × 7 × 5 and 3 × 11 × 7 grid, respectively. Self-consistent-field convergence criterion was set to 10^–7^ eV. Additionally at this level, dynamical stability of resulting structures was assessed by calculating phonon frequencies at Gamma point of the Brillouin zone. Structures were further optimized to minimize forces acting on atoms to ≤ 10^–5^ eV/Å, and then Hessian matrix was constructed through finite differences approach as implemented in VASP. Phonon dispersion curves for selected systems were calculated and generated using PHONOPY interface^34,35^. PBEsol + U is the cheapest among the three methods, but has been successfully utilized for prediction of high-pressure structures of Ag(II) compounds in the past^2–4,36^.
II. HSE06 approach: Hybrid-DFT HSE06 functional was utilized^37^. Due to higher computational load of this method, a coarser k-space sampling of ca. 2π × 0.05 Å^−1^ was used (e.g. 4 × 4 × 3 grid in AgF_2_-type) and plane-wave cut-off was set to 520 eV, with self-consistent-field convergence criterion of 10^–7^ eV. Although the cutoff energy is lower than in the other two methods, our convergence tests have shown that increasing it to e.g. 600 eV does not lead to a substantial improvement (i.e. the resulting total energy change is ca. 0.0001 eV/FU, which is of no practical consequence in our data). HSE06 is the most computationally demanding of the three methods, but provides the most accurate description of electronic properties^19^.
III. SCAN approach: Meta-GGA-type, strongly constrained and appropriately normed (SCAN) functional was used^38^, with correction for van der Waals interactions^39^. Plane-wave cut-off energy was set to 800 eV and k-space sampling of ca. 2π × 0.04 Å^−1^ was used, with self-consistent-field convergence criterion of 5 × 10^–7^ eV. A denser k-spacing of ca. 2π × 0.03 Å^−1^ was used for eDOS calculations. SCAN approach is slightly more expensive than PBEsol + U, but still much cheaper than HSE06. This method has previously provided a value of antiferromagnetic coupling constant in AgF_2_ that is in best agreement with experimental data^2^.

The studied pressure range was 0 to 60 gigapascals (GPa), additionally extended to 100 GPa for SCAN calculations. Integration grids were not substantially increased in metallic solutions at higher pressures, but our convergence tests showed that an increase of grid density does not lead to a meaningful change in total energy (within 0.005 eV/FU) or in lattice constants after re-optimization (within 0.01 Å) at denser mesh. Pressure step was 10 GPa up to 60 GPa (or in some cases 15 GPa in HSE06 approach) and 20 GPa above 60 GPa. Cl_2_ was considered in its solid polymorph with *Cmca* space group, which is known to be stable in the entire pressure range considered here^40^. Similarly, metallic silver is also stable in its fcc structure within that range^41^. Known phase transitions of AgCl were taken into account when calculating relative enthalpy of AgCl_2_ polymorphs^42^. A primitive unit cell of the KOH-type, high-pressure polymorph of AgCl was utilized in calculations, since it can be used to accurately describe the continuous nature of NaCl–KOH–TlI–CsCl sequence of phase transitions of AgCl^42,43^.

## Supplementary Information

Supplementary Information.
