# Supplementary material for: Stability of hypothetical AgIICl2 polymorphs under high pressure, revisited: a computational study
Source: Sci Rep. 2022 Jan 21;12:1153. doi: 10.1038/s41598-022-05211-0 (PMC8782826; doi:10.1038/s41598-022-05211-0)
Supplement: Supplementary file 1 — Supplementary Information. [file 41598_2022_5211_MOESM1_ESM.docx]

Stability of hypothetical Ag^II^Cl_2_ polymorphs under high pressure, revisited – a computational study

*Adam Grzelak^1^* and Wojciech Grochala^1^*

*^1^Center for New Technologies, University of Warsaw, Banacha 2C, 02-097 Warszawa, Poland*

**a.grzelak@cent.uw.edu.pl*

SUPPLEMENTARY INFORMATION

**Contents**

[1. Phonon dispersion curves obtained for selected polymorphs within PBEsol+U approach 3](#_Toc89167443)

[Ag(I)r, 0 GPa, 2x2x2 supercell 3](#_Toc89167444)

[CuCl_2_, 0 GPa, 1x2x2 supercell 3](#_Toc89167445)

[2. Structures of studied AgCl_2_ polymorphs at 0 GPa in VASP (POSCAR) format 4](#_Toc89167446)

[Ag(I)h, PBEsol+U 4](#_Toc89167447)

[Ag(I)r, PBEsol+U 4](#_Toc89167448)

[AgF_2_, PBEsol+U 4](#_Toc89167449)

[AuCl_2_, PBEsol+U 5](#_Toc89167450)

[AuCl_2_, HSE06 5](#_Toc89167451)

[CuCl_2_, PBEsol+U 5](#_Toc89167452)

[MnO_2_, PBEsol+U 6](#_Toc89167453)

[HP-AgF_2_, PBEsol+U 6](#_Toc89167454)

[3. Selected structures at higher pressures and from different methods in VASP (POSCAR) format 7](#_Toc89167455)

[CuCl_2_, 20 GPa, PBSEsol+U 7](#_Toc89167456)

[CuCl_2_, 20 GPa, SCAN 7](#_Toc89167457)

[AgF_2_, 30 GPa, PBEsol+U 8](#_Toc89167458)

[AgF2_2_, 40 GPa, PBEsol+U 8](#_Toc89167459)

[AgF_2_, 40 GPa, HSE06 8](#_Toc89167460)

[Ag(I)r, 60 GPa, PBEsol+U 9](#_Toc89167461)

[HP-AgF_2_, 60 GPa, PBEsol+U 9](#_Toc89167462)

[HP-AgF_2_, 60 GPa, HSE06 10](#_Toc89167463)

[AuCl_2_, 20 GPa, PBEsol+U 10](#_Toc89167464)

[AuCl_2_, 30 GPa, PBEsol+U 11](#_Toc89167465)

[AuCl_2_, 40 GPa, PBEsol+U 11](#_Toc89167466)

[AuCl_2_, 30 GPa, HSE06 11](#_Toc89167467)

[AuCl_2_, 30 GPa, SCAN 12](#_Toc89167468)

[AuCl_2_, 60 GPa, SCAN 12](#_Toc89167469)

[4. Selected structures of phonon-relaxed AgF_2_- and HP-AgF_2_-type polymorphs in VASP (POSCAR) format 13](#_Toc89167470)

[AgF_2_ phonon-relaxed, 10 GPa, PBEsol+U 13](#_Toc89167471)

[AgF_2_ phonon-relaxed, 30 GPa, PBEsol+U 13](#_Toc89167472)

[AgF_2_ phonon-relaxed, 50 GPa, PBEsol+U 13](#_Toc89167473)

[HP-AgF_2_ phonon-relaxed, 0 GPa, PBSEsol+U 14](#_Toc89167474)

[HP-AgF_2_ phonon-relaxed, 20 GPa, PBSEsol+U 14](#_Toc89167475)

[HP-AgF_2_ phonon-relaxed, 30 GPa, PBSEsol+U 15](#_Toc89167476)

[HP-AgF_2_ phonon-relaxed, 40 GPa, PBSEsol+U 16](#_Toc89167477)

# 1. Phonon dispersion curves obtained for selected polymorphs within PBEsol+U approach

Phonon dispersion curves were calculated using settings described for PBEsol+U in “Methods” section in the main text.

## Ag(I)r, 0 GPa, 2x2x2 supercell


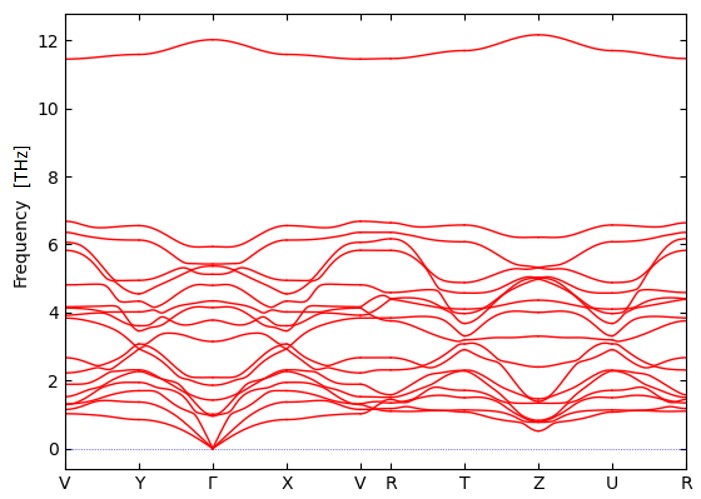


## CuCl_2_, 0 GPa, 1x2x2 supercell


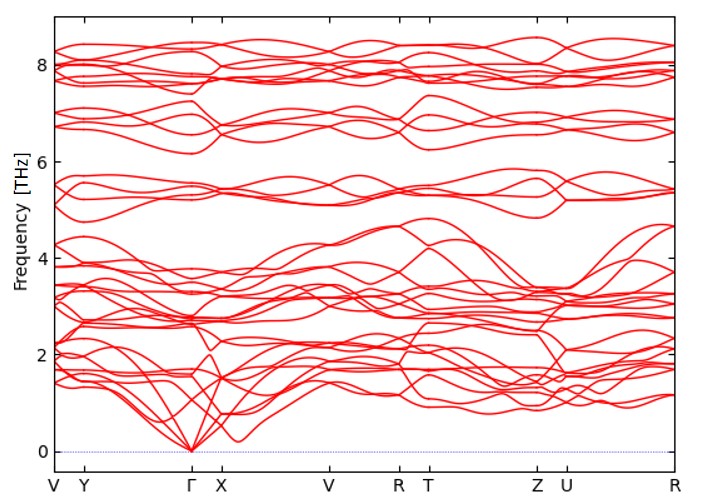


# 2. Structures of studied AgCl_2_ polymorphs at 0 GPa in VASP (POSCAR) format

## Ag(I)h, PBEsol+U

Ag(I)h

1.00000000000000

4.1809389435423832 0.0000737301493584 -0.0002449425727769

-2.0739920604287132 3.9321185474036251 -0.0001700360877051

-0.0438825227124686 -1.6701279908281432 9.3968745559511557

Ag Cl

2 4

Direct

0.6358915517804045 0.7730362273299862 0.8563886066398888

0.3641084682195972 0.2269638256700146 0.1436114523601125

0.4450575397368848 0.3916670961301965 0.5904152633794647

0.5549425042631188 0.6083328898698025 0.4095847836205357

0.3089962195205326 0.1223607080926478 0.8516426717795432

0.6910037834794711 0.8776393679073514 0.1483573362204574

## Ag(I)r, PBEsol+U

Ag(I)r

1.00000000000000

3.7724821451995476 -0.0001704139084030 -0.0002754981947617

0.0244296040738955 3.7749682901047015 -0.0005130426459340

1.0500398576570700 1.0559751440702208 9.1444928727785424

Ag Cl

2 4

Direct

0.2749641719255636 0.2767721603938008 0.8520390410506722

0.7250358140744352 0.7232278036062033 0.1479609709493288

0.5812454366217329 0.5815468551596583 0.5937159417203364

0.4187545213782706 0.4184531438403379 0.4062840562796634

0.7912169444577231 0.7926909967264482 0.8543772747008583

0.2087830325422716 0.2073089732735493 0.1456227112991406

## AgF_2_, PBEsol+U

AgF2

1.00000000000000

5.8576235214886037 -0.0000000000000003 0.0000000000000000

0.0000000000000000 6.1994368733598408 0.0000000000000000

0.0000000000000000 0.0000000000000000 6.9335916976853120

Ag Cl

4 8

Direct

0.0000000000000000 -0.0000000000000000 -0.0000000000000000

0.5000000000000000 0.0000000000000000 0.5000000000000000

-0.0000000000000000 0.5000000000000000 0.5000000000000000

0.5000000000000000 0.5000000000000000 -0.0000000000000000

0.1636720499546089 0.1736595265749634 0.3718035028199924

0.8363279390453866 0.8263404954250384 0.6281964561800076

0.3363279390453866 0.8263404954250384 0.8718035438199924

0.6636720609546134 0.1736595265749634 0.1281964871800068

0.8363279390453866 0.6736595475749616 0.1281964871800068

0.1636720499546089 0.3263404524250385 0.8718035438199924

0.6636720609546134 0.3263404524250385 0.6281964561800076

0.3363279390453866 0.6736595475749616 0.3718035028199924

## AuCl_2_, PBEsol+U

AuCl2

1.00000000000000

6.3800164859980244 0.3750280089695290 0.1984250261055866

-1.2996383865542038 6.1122404529775674 -0.0315137536090649

-2.2062471169903262 -1.3356193827024136 6.6427810249038366

Ag Cl

4 8

Direct

0.9669655584420562 0.7338284713455612 0.8624677465890955

0.0330343995579475 0.2661715336544360 0.1375322004109040

0.4614533304817907 0.9885836254403092 0.7422865415725698

0.5385466285182094 0.0114163965596852 0.2577133974274284

0.1842560374836626 0.6647379189411198 0.6356885755115412

0.8157438585163364 0.3352620910588809 0.3643113944884568

0.3206431418550906 0.2051411992894391 0.4205776499143179

0.6793568531449127 0.7948588227105698 0.5794222880856698

0.2455491804669422 0.1623659767903010 0.9159449382000595

0.7544507925330590 0.8376340202097020 0.0840550557999399

0.7452188651113193 0.3180063334286093 0.8471930503383647

0.2547811768886769 0.6819936615713865 0.1528069356616347

## AuCl_2_, HSE06

AuCl2

1.00000000000000

7.7992974392019088 -0.7493790403714657 0.7720990416449384

-0.0198469543274694 6.3817833303945974 0.1605930039235957

3.0789604775335135 -0.1331976352862337 6.5499577868900367

Ag Cl

4 8

Direct

0.1531757314680115 0.7492576021043830 0.0492125649074180

0.8468242685319886 0.2507423978956099 0.9507874570925767

0.2413342592154223 0.9147064848864621 0.5019871135382864

0.7586657557845754 0.0852935151135379 0.4980128864617135

0.3839002906808242 0.7416780241602572 0.1497970948268173

0.6160997383191746 0.2583219758397429 0.8502029051731826

0.5955588890776555 0.2336872231556989 0.3034014472344190

0.4044411109223445 0.7663127918443059 0.6965985677655859

0.0752231432354336 0.2520970384559839 0.0576916968525954

0.9247768567645733 0.7479029465440115 0.9423083331473999

0.0893384215859799 0.2569385723249594 0.5938865923268923

0.9106615934140178 0.7430614566750393 0.4061134076731077

## CuCl_2_, PBEsol+U

CuCl2

1.00000000000000

14.0979550310688797 0.0369242398089084 -0.1515180561896840

-1.7506072931762209 3.4784929972500827 0.1035297901793503

-0.0828159240334310 -0.8706943205567950 5.3263190753779872

Ag Cl

4 8

Direct

0.2502907755323037 0.4993863901490141 0.5019840786879673

0.4997092400997359 0.5006136679805866 0.4980159908320100

0.7502907755323037 0.4993863901490141 0.5019840786879673

0.9997092440997363 0.5006136679805866 0.4980159908320100

0.0846720831744846 0.1919935184559102 0.2363151246817081

0.3337559342444317 0.1905389257430200 0.2369233735662472

0.5846721121744833 0.1919935184559102 0.2363151246817081

0.8337559672444309 0.1905389257430200 0.2369233735662472

0.1653279273326591 0.8080065386674057 0.7636849428672593

0.4162440416163781 0.8094611330040777 0.7630766953647968

0.6653279273326591 0.8080065386674057 0.7636849428672593

0.9162441086163801 0.8094611330040777 0.7630766953647968

## MnO_2_, PBEsol+U

MnO2

1.00000000000000

3.9277614743228910 0.0000000000000000 0.0197958681961648

0.0000000000000000 11.5198076245511629 0.0000000000000000

-3.2921591218614505 0.0000000000000000 5.8942457665163976

Ag Cl

4 8

Direct

0.2724530553403969 0.1295003731277268 0.5769166071930415

0.7275469666596053 0.8704995858722735 0.4230834138069567

0.7275469476596002 0.6295003731277267 0.9230834238069575

0.2724530693403981 0.3704996268722732 0.0769166621930425

0.5615698945328782 0.2453524905848668 0.9136630174980422

0.4384301444671215 0.7546475094151336 0.0863370225019540

0.4384301684671164 0.7453524905848664 0.5863370125019531

0.5615698495328780 0.2546475094151332 0.4136629674980452

0.0012439053343415 0.9996651102090802 0.2527967684561538

-0.0012438833343397 0.0003349287909194 0.7472032515438480

-0.0012438943343442 0.4996650692090804 0.2472032415438471

0.0012439503343417 0.5003348897909199 0.7527968184561507

## HP-AgF_2_, PBEsol+U

HP-AgF2

1.00000000000000

8.4465644144972938 0.0000000000000000 0.0000000000000000

0.0000000000000000 9.5680214472431100 0.0000000000000000

0.0000000000000000 0.0000000000000000 6.2837058901439971

Ag Cl

8 16

Direct

0.6672118589315235 0.6068237947390006 0.4414822640720134

0.3327881220684787 0.3931762322609982 0.5585177359279867

0.8327881410684765 0.8931762052609994 0.9414822640720133

0.1672119379315193 0.1068237677390019 0.0585177449279838

0.3327881220684787 0.6068237947390006 0.0585177449279838

0.6672118589315235 0.3931762322609982 0.9414822640720133

0.1672119379315193 0.8931762052609994 0.5585177359279867

0.8327881410684765 0.1068237677390019 0.4414822640720134

0.8212509752791749 0.5658088784653912 0.1204008867153574

0.1787490247208248 0.4341910955346144 0.8795991322846407

0.6787490247208251 0.9341911215346088 0.6204008677153593

0.3212509752791752 0.0658089044653858 0.3795991322846407

0.1787490247208247 0.5658088784653912 0.3795991322846407

0.8212509752791749 0.4341910955346144 0.6204008677153593

0.3212509752791752 0.9341911215346088 0.8795991322846407

0.6787490247208251 0.0658089044653858 0.1204008867153574

0.5000000000000000 0.6359079300369206 0.7500000000000000

0.5000000000000000 0.3640920969630785 0.2500000000000000

0.0000000000000000 0.8640920699630794 0.2500000000000000

0.0000000000000000 0.1359078500369209 0.7500000000000000

0.5000000000000000 0.2369243841107499 0.7500000000000000

0.5000000000000000 0.7630755898892477 0.2500000000000000

0.0000000000000000 0.2630756158892503 0.2500000000000000

1. 7523 0.7500000000000000

# 3. Selected structures at higher pressures and from different methods in VASP (POSCAR) format

## CuCl_2_, 20 GPa, PBSEsol+U

CuCl2

1.00000000000000

14.7080063977111966 -0.0406361621980564 0.0607430204407703

-1.8332441531448587 2.4693030577957580 -0.4996373026560079

-0.0217539356240984 -1.5228353225210398 5.3698064122627303

Ag Cl

4 8

Direct

0.2499972963967593 0.4999981703690119 0.5000009134728353

0.5000027192352803 0.5000018877605886 0.4999991560471422

0.7499972963967591 0.4999981703690119 0.5000009134728353

0.0000027232352808 0.5000018877605886 0.4999991560471422

0.0873466593439202 0.1982822742158889 0.1963152786819687

0.3373475960705180 0.1982749256051968 0.1963082084068231

0.5873466883439261 0.1982822742158890 0.1963152786819687

0.8373476290705172 0.1982749256051968 0.1963082084068231

0.1626533511632234 0.8017177829074268 0.8036847888669988

0.4126523797902918 0.8017251331419011 0.8036918605242205

0.6626533511632163 0.8017177829074268 0.8036847888669988

0.9126524467902938 0.8017251331419011 0.8036918605242205

## CuCl_2_, 20 GPa, SCAN

CuCl2

1.00000000000000

13.0049236743343908 3.8758808916381349 -2.0254818978709457

-0.8300396270216698 3.0397017923566234 0.3370997964913102

-0.8292354921452237 -0.8649866089139385 4.7326375831941894

Ag Cl

4 8

Direct

0.2500124393280386 0.5001208580380111 0.5000254172640733

0.4999875763040011 0.4998792000915896 0.4999746522559033

0.7500124393280388 0.5001208580380111 0.5000254172640733

-0.0000124196959986 0.4998792000915896 0.4999746522559033

0.0871482751298815 0.2091347449362864 0.1958180870219174

0.3371231481482379 0.2091915825029764 0.1957995825896365

0.5871483041298809 0.2091347449362864 0.1958180870219174

0.8371231811482374 0.2091915825029764 0.1957995825896365

0.1628517353772621 0.7908653121870297 0.8041819805270501

0.4128768277125719 0.7908084762441214 0.8042004863414075

0.6628517353772615 0.7908653121870297 0.8041819805270501

0.9128768947125736 0.7908084762441214 0.8042004863414075

## AgF_2_, 30 GPa, PBEsol+U

AgF2

1.00000000000000

5.6698461580421444 -0.0000000000290830 0.0000000000000000

-0.0000000000298380 5.8191414994981692 0.0000000000000000

0.0000000000000000 0.0000000000000000 5.6409531037753444

Ag Cl

4 8

Direct

-0.0000000000000000 0.0000000000000000 -0.0000000000000000

0.5000000000000000 0.0000000000000000 0.5000000000000000

-0.0000000000000000 0.5000000000000000 0.5000000000000000

0.5000000000000000 0.5000000000000000 -0.0000000000000000

0.1461531626584120 0.1547762644201493 0.3564766877460681

0.8538468263415836 0.8452237575798526 0.6435232712539318

0.3538468263415835 0.8452237575798526 0.8564767287460682

0.6461531736584164 0.1547762644201493 0.1435233022539310

0.8538468263415846 0.6547762854201461 0.1435233022539310

0.1461531626584109 0.3452237145798541 0.8564767287460682

0.6461531736584154 0.3452237145798541 0.6435232712539318

0.3538468263415846 0.6547762854201461 0.3564766877460681

## AgF2_2_, 40 GPa, PBEsol+U

AgF2

1.00000000000000

4.7366565397196956 -0.0000004392386906 0.0000000000000000

-0.0000008854746414 9.5581537843213003 0.0000000000000000

0.0000000000000000 0.0000000000000000 3.6126147470587693

Ag Cl

4 8

Direct

0.0000000000000000 0.0000000000000000 0.0000000000000000

0.5000000000000000 0.0000000000000000 0.5000000000000000

0.0000000000000000 0.5000000000000000 0.5000000000000000

0.5000000000000000 0.5000000000000000 0.0000000000000000

-0.0000542882231508 0.1550463477209816 0.5000236940429762

0.0000542772231460 0.8449536742790204 0.4999762649570241

0.5000542772231464 0.8449536742790205 0.0000237350429762

0.4999457227768542 0.1550463477209816 -0.0000237040429770

0.0000542772163547 0.6550463687975343 -0.0000237040429770

-0.0000542882163597 0.3449536312024657 0.0000237350429762

0.4999457227836454 0.3449536312024657 0.4999762649570240

0.5000542772163550 0.6550463687975343 0.5000236940429763

## AgF_2_, 40 GPa, HSE06

AgCl2 AgF2

1.00000000000000

4.0554222842139538 -0.0033160166740209 0.0000000000000000

-0.0037832666121039 4.5945927105887163 0.0000000000000000

0.0000000000000000 0.0000000000000000 9.2277860949152135

Ag Cl

4 8

Direct

0.0000000000000000 0.0000000000000000 -0.0000000000000000

0.5000000000000000 0.0000000000000000 0.5000000000000000

0.0000000000000000 0.5000000000000000 0.5000000000000000

0.5000000000000000 0.5000000000000000 -0.0000000000000000

0.0248260389927012 0.0931842314325581 0.3471323932680363

-0.0248260499927059 0.9068157905674440 0.6528675657319641

0.4751739500072942 0.9068157905674440 0.8471324342680359

0.5248260499927059 0.0931842314325581 0.1528675967319628

-0.0248269075477147 0.5931845185304153 0.1528675967319628

0.0248268965477103 0.4068154814695851 0.8471324342680359

0.5248269075477143 0.4068154814695850 0.6528675657319641

0.4751730924522854 0.5931845185304153 0.3471323932680362

## Ag(I)r, 60 GPa, PBEsol+U

Ag(I)r

1.00000000000000

2.8284324608546298 0.1356947984556557 -0.3416285111821801

0.1942495520457748 4.2179025425351719 0.2987026501038441

0.0259062706300690 2.1218693103105002 6.5032535679628571

Ag Cl

2 4

Direct

0.3693396435131028 0.3245004130744808 0.8949445201928918

0.6306603424868960 0.6754995509255233 0.1050554918071090

0.6381256340654331 0.6486514986324431 0.5700094933982898

0.3618743239345703 0.3513485003675532 0.4299905046017103

-0.0159280193170598 0.9247550786540184 0.7822871073388831

0.0159279963170544 0.0752448913459792 0.2177128786611162

## HP-AgF_2_, 60 GPa, PBEsol+U

HP-AgF2

1.00000000000000

4.9076039785685195 0.0000000000000000 -0.0000000000000006

0.0000000000000000 8.6818101603758890 0.0000000000000000

-0.0000000000000009 0.0000000000000000 7.0073518224603797

Ag Cl

8 16

Direct

0.6928483116196761 0.6203101842937221 0.4125947899896464

0.3071516693803255 0.3796898427062765 0.5874052100103534

0.8071516883803239 0.8796898157062779 0.9125947899896466

0.1928483906196723 0.1203101572937233 0.0874052190103508

0.3071516693803255 0.6203101842937222 0.0874052190103508

0.6928483116196761 0.3796898427062765 0.9125947899896466

0.1928483906196723 0.8796898157062778 0.5874052100103534

0.8071516883803239 0.1203101572937233 0.4125947899896465

0.8206496221659293 0.6276355943900015 0.0799042453346620

0.1793503778340707 0.3723643796100033 0.9200957736653359

0.6793503778340707 0.8723644056099985 0.5799042263346641

0.3206496221659292 0.1276356203899965 0.4200957736653361

0.1793503778340707 0.6276355943900015 0.4200957736653361

0.8206496221659293 0.3723643796100033 0.5799042263346641

0.3206496221659292 0.8723644056099985 0.9200957736653359

0.6793503778340707 0.1276356203899965 0.0799042453346620

0.5000000000000000 0.6106700341354168 0.7500000000000000

0.5000000000000000 0.3893299928645817 0.2500000000000000

0.0000000000000000 0.8893299658645832 0.2500000000000000

0.0000000000000000 0.1106699541354174 0.7500000000000000

0.5000000000000000 0.1381817620179306 0.7500000000000000

0.5000000000000000 0.8618182119820672 0.2500000000000000

0.0000000000000000 0.3618182379820694 0.2500000000000000

0.0000000000000000 0.6381817880179329 0.7500000000000000

## HP-AgF_2_, 60 GPa, HSE06

HP-AgF2

1.00000000000000

5.7213137892555492 0.0000000000000000 -0.0000001716442535

0.0000000000000000 8.6425975247664795 0.0000000000000000

-0.0000001920398294 0.0000000000000000 6.4035796677243084

Ag Cl

8 16

Direct

0.6839950216202747 0.6196666946849928 0.4293982622599136

0.3160049593797198 0.3803333323149989 0.5706017377400866

0.8160049783798713 0.8803333053150072 0.9293982622581803

0.1839951006201247 0.1196666676849940 0.0706017467418167

0.3160049593797198 0.6196666946849928 0.0706017467400837

0.6839950216202747 0.3803333323149989 0.9293982622599134

0.1839951006201247 0.8803333053150072 0.5706017377418197

0.8160049783798713 0.1196666676849940 0.4293982622581805

0.8233576064139391 0.5927464137800074 0.0950788641962084

0.1766423935860608 0.4072535602199977 0.9049211548037894

0.6766423935855225 0.9072535862199926 0.5950788451956061

0.3233576064144775 0.0927464397800022 0.4049211548043937

0.1766423935860608 0.5927464137800074 0.4049211548037896

0.8233576064139391 0.4072535602199977 0.5950788451962106

0.3233576064144775 0.9072535862199926 0.9049211548043939

0.6766423935855225 0.0927464397800022 0.0950788641956042

0.5000000000000000 0.6423529188902207 0.7500000000000000

0.5000000000000000 0.3576471081097778 0.2500000000000000

-0.0000000000000000 0.8576470811097793 0.2500000000000000

-0.0000000000000000 0.1423528388902215 0.7500000000000000

0.5000000000000000 0.1850647416854350 0.7500000000000000

0.5000000000000000 0.8149352323145628 0.2500000000000000

-0.0000000000000000 0.3149352583145649 0.2500000000000000

-0.0000000000000000 0.6850647676854372 0.7500000000000000

## AuCl_2_, 20 GPa, PBEsol+U

AuCl2

1.00000000000000

5.8910763497432237 0.4095921217242142 0.3444311862858598

-1.1656297350202272 5.5741279026053672 0.6704393710303472

-1.9448402931624291 -0.3516373719228075 5.4990055270764016

Ag Cl

4 8

Direct

0.0656508416378020 0.7157374536671796 0.8460393727658539

-0.0656508836377985 0.2842625513328171 0.1539605742341455

0.5327577724241086 0.9495809111724911 0.7951479165852737

0.4672421865758923 0.0504191108275033 0.2048520224147249

0.2467651289205502 0.6424269487082130 0.5719279570994574

0.7532347670794479 0.3575730612917884 0.4280720129005398

0.2322610355216878 0.1554505210386258 0.4600613538354951

0.7677389594783154 0.8445495009613833 0.5399385841644931

0.1218021457705002 0.2092207985280528 0.8819186456698678

0.8781978272295015 0.7907791984719511 0.1180813483301246

0.6144068080897772 0.3749890078937723 0.8453752113926482

0.3855932339102189 0.6250109871062238 0.1546247746073505

## AuCl_2_, 30 GPa, PBEsol+U

AuCl2

1.00000000000000

5.2388807570425460 0.7591387508223031 -0.0120295582117378

-0.7573508152388195 4.4655850395806400 0.7181920590674146

-2.2310097768416641 -0.6935649853665323 7.1907910903615058

Ag Cl

4 8

Direct

0.1631326735092760 0.5800703394701714 0.8045024996780469

0.8368672844907278 0.4199296655298257 0.1954974473219522

0.6040373167654201 0.9886847971123240 0.7995339453270333

0.3959626422345804 0.0113152248876707 0.2004659936729653

0.2521099190878114 0.6233918721609149 0.5018279957530943

0.7478899769121873 0.3766081378390857 0.4981719742469031

0.2439369011613220 0.1293899379628367 0.4897435321643449

0.7560630938386813 0.8706100840371722 0.5102564058356432

0.1504283286038952 0.0702521628552519 0.8544275737817860

0.8495716443961061 0.9297478341447513 0.1455724202182063

0.6922570138834885 0.4801752073095487 0.8571722893201356

0.3077430281165078 0.5198247876904470 0.1428276966798634

## AuCl_2_, 40 GPa, PBEsol+U

AuCl2

1.00000000000000

5.8082520951364529 1.0898519928949033 0.2105567687986048

-0.5587599145336952 4.1587278109242041 0.9416948466951989

-2.1510052685533823 -0.3306661032465344 6.5509673985961587

Ag Cl

4 8

Direct

0.0074565472700761 0.7825924870445131 0.8932421873827048

-0.0074565892700724 0.2174075179554841 0.1067577596172949

0.5077423806164141 0.7823819056284268 0.8933874467067825

0.4922575783835865 0.2176181163715676 0.1066124922932159

0.2158093311415543 0.7848433348901015 0.5703014052754537

0.7841905648584445 0.2151566751099002 0.4296985647245448

0.2843171347843280 0.2156937039068180 0.4299035402982176

0.7156828602156747 0.7843063180931911 0.5700963977017702

0.1514856042452872 0.2954227380063346 0.7891578835953472

0.8485143687547141 0.7045772589936690 0.2108421104046452

0.6516492916321169 0.2949784809123317 0.7893226446716651

0.3483507503678800 0.7050215140876648 0.2106773413283334

## AuCl_2_, 30 GPa, HSE06

AuCl2

1.00000000000000

6.8065614038350244 -1.8760548875834224 1.2677229308869149

-0.9058388423465875 5.8197160080270383 0.6010925086192211

3.1124389597131636 -0.0364483403912550 5.5428754701286209

Ag Cl

4 8

Direct

0.2102803227132909 0.7541210029256751 -0.0185734201951061

0.7897196772867094 0.2458789970743175 0.0185734421951010

0.2201024521456740 0.9523391506222683 0.4703510423299078

0.7798975628543237 0.0476608493777324 0.5296489576700919

0.5286220336615437 0.7602482125316800 -0.0017270294309441

0.4713779953384555 0.2397517874683199 0.0017270294309439

0.5363771857292431 0.2685671756051204 0.4812428917643077

0.4636228142707567 0.7314328393948842 0.5187571232356961

0.1103817448767674 0.2551532293913981 0.0268779634299011

0.8896182551232393 0.7448467556085964 0.9731220665700946

0.1057929545409776 0.3448813736105729 0.5317569588496148

0.8942070604590203 0.6551186553894256 0.4682430411503848

## AuCl_2_, 30 GPa, SCAN

AuCl2

1.00000000000000

5.8687230275696649 -0.5292533647186523 -0.1092707456119816

-0.4180224470103834 5.7700121711612828 0.3941673652513003

-1.5624667802462568 0.2975157558775671 5.5998613229336298

Ag Cl

4 8

Direct

0.5431984189608760 0.4605684971484660 0.2944104086536523

0.4568016090391260 0.5394314668515310 0.7055895463463472

0.0669918754722515 0.2175973778543996 0.3421451065169002

-0.0669918334722479 0.7824025871456012 0.6578548664831019

0.2483731776148961 0.1467898467052986 0.0711832051543068

0.7516267953851050 0.8532100802946988 0.9288167298456912

0.3853720924466498 0.1200768588290079 0.6517763651384136

0.6146279425533497 0.8799230711709932 0.3482236078615877

0.8826668298222690 0.2884158589088387 0.6164840425217154

0.1173332221777281 0.7115840880911605 0.3835159314782819

0.7664815885801457 0.3399489633391335 0.0357032750957524

0.2335184684198552 0.6600510166608646 0.9642966609042419

## AuCl_2_, 60 GPa, SCAN

AuCl2

1.00000000000000

4.6813862764959984 -0.6637229353822505 -0.3981220069615501

-0.4399980797143317 5.5115825663583111 0.5393590554555442

-1.3752343662312372 0.4769575471437021 6.4360749066978631

Ag Cl

4 8

Direct

0.6047728610542724 0.4954170440800851 0.3168447489613741

0.3952271669457299 0.5045829199199122 0.6831552060386251

0.1001010706192541 0.2295236698671017 0.3206200243917104

-0.1001010286192506 0.7704762951328989 0.6793799486082911

0.2090297448435288 0.1045217490138356 0.0227307454486402

0.7909702281564720 0.8954781779861622 0.9772691895513574

0.3447311592566148 0.0408859177294480 0.6474175666547245

0.6552688757433841 0.9591140122705537 0.3525824063452767

0.8658070804783718 0.3141590750276319 0.6497104679005826

0.1341929715216250 0.6858408719723680 0.3502895060994154

0.7133889414740744 0.3564719108222293 0.0162902041970780

0.2866111155259270 0.6435280691777685 0.9837097318029167

# 4. Selected structures of phonon-relaxed AgF_2_- and HP-AgF_2_-type polymorphs in VASP (POSCAR) format

## AgF_2_ phonon-relaxed, 10 GPa, PBEsol+U

AgF2

1.00000000000000

5.6397429056665782 -0.0000572947421523 0.0000066457862113

-0.0001466141089493 5.9704507900963071 0.0000000051871313

0.0000074720551636 0.0000000051112625 6.3341294004102142

Ag Cl

4 8

Direct

0.0174065021504977 0.9606439099721849 0.0205987942555129

0.4825935011538640 0.0393561150281507 0.5205987930674979

0.0173822100036346 0.5393558854363344 0.5205934242695116

0.4826177932875867 0.4606440845640229 0.0205934250861107

0.1555189209207009 0.2079592478280732 0.3482398976189029

0.8464754576075232 0.8792763842450866 0.6283523669715499

0.3444810789414527 0.7920407961715991 0.8482399575751615

0.6535245422530187 0.1207236447548554 0.1283524269562894

0.8464572838766118 0.6207300818061057 0.1283350445328897

0.1555136920251864 0.2920236504251356 0.8482375836059826

0.6535427159677880 0.3792699181936928 0.6283349845054088

0.3444863078121359 0.7079763495747651 0.3482375235551811

## AgF_2_ phonon-relaxed, 30 GPa, PBEsol+U

AgF2

1.00000000000000

5.1868945502740962 -0.0016136149059700 -0.0002733453878625

0.0003438278362833 6.0318148307575354 -0.0022654236549465

0.0053551503626217 0.0022595118236022 5.7121271803074984

Ag Cl

4 8

Direct

0.0411075785704253 0.9278479825460361 0.0022410870803859

0.4592376187479439 0.0721874983771948 0.5014123442242920

0.0410272453672045 0.5722131474326341 0.6634883849990283

0.4585950710327524 0.4278526139361821 0.1626234490244223

0.0983946151484483 0.2497955100914500 0.3324141942412633

0.8502524810058137 0.9026120250068423 0.5563809596809125

0.4016380195663773 0.7501507573279222 0.8323810510332379

0.6498605636285039 0.0975631558775332 0.0558000046318598

0.8499940911120955 0.5973347589678000 0.1089886905248390

0.1882927988960926 0.2498559867561827 0.8321649647137228

0.6499355840372500 0.4028002267372275 0.6082915316081805

0.3116643388870933 0.7497864049429930 0.3325275602378475

## AgF_2_ phonon-relaxed, 50 GPa, PBEsol+U

AgF2

1.00000000000000

4.5200886725630962 -0.0045426260690987 -0.0008532688638541

0.0084498756289119 7.5965040727758204 -0.0002904597084789

0.0020542438851857 0.0013823844847637 4.5585617534714400

Ag Cl

4 8

Direct

-0.0000721916061678 0.9183568549842058 0.1335663888089586

0.5000678630995368 0.0816160242772641 0.5309266894952756

-0.0000538157983574 0.5815922498942161 0.6342552542594219

0.4999911333416494 0.4184825086294140 0.0308544027231043

-0.0001832776226515 0.2383890901801509 0.3118169970430116

0.0001456524896029 0.9136908470980658 0.6385850757224155

0.5001941221669707 0.7383740253817814 0.8528939523049925

0.4997379649756160 0.0862830764983408 0.0259148708407180

0.0003296454148932 0.5863882525220772 0.1392714243362007

-0.0001937065098825 0.2616179630264345 0.8118793860887540

0.4998476947226168 0.4135738828568103 0.5258233950116588

0.5001889213261671 0.7616352926512364 0.3529263853654871

## HP-AgF_2_ phonon-relaxed, 0 GPa, PBSEsol+U

HP-AgF2

1.00000000000000

8.5479047664156589 0.4006668401954080 0.0000000000000000

0.4615187391631804 9.5100885483570554 0.0000000000000000

0.0000000000000000 0.0000000000000000 6.2589864004402029

Ag Cl

8 16

Direct

0.6373406642380957 0.6300862313754975 0.4500146947933866

0.3626593057619025 0.3699137686245025 0.5499853352066157

0.8626593357619043 0.8699137686245025 0.9500146647933843

0.1373407392380978 0.1300862313754975 0.0499853202066183

0.3002183474896988 0.5829004212630776 0.0572015694312778

0.6997816525103013 0.4170996087369179 0.9427984345687227

0.1997817115103024 0.9170995787369223 0.5572015654312773

0.8002183474896987 0.0829003912630820 0.4427984345687226

0.8028963813627223 0.6100972391585133 0.1301333566404548

0.1971036036372797 0.3899027308414918 0.8698666733595403

0.6971036186372777 0.8899027608414867 0.6301333266404597

0.3028963813627228 0.1100972761585126 0.3698666733595406

0.1604191030103701 0.5110378290694413 0.3653512695010261

0.8395808669896272 0.4889621409305560 0.6346487004989788

0.3395808969896297 0.9889621709305587 0.8653512995010212

0.6604191330103728 0.0110378470694430 0.1346487454989790

0.4637814612080510 0.6289803481953743 0.7572015027654978

0.5362185387919490 0.3710196518046261 0.2427984822345045

0.0362185347919487 0.8710196518046257 0.2572015027654977

0.9637814612080510 0.1289802891953726 0.7427984972345022

0.5760697914322854 0.2442204172737438 0.7397030387411486

0.4239302085677147 0.7557795227262587 0.2602969612588518

0.9239302085677148 0.2557795827262564 0.2397030387411486

0.0760697824322882 0.7442204772737413 0.7602969612588514

## HP-AgF_2_ phonon-relaxed, 20 GPa, PBSEsol+U

HP-AgF2

1.00000000000000

6.3394068150579237 0.0348541866128099 0.0000000000000000

-0.0510814320277891 9.3251079280373315 0.0000000000000000

0.0000000000000000 0.0000000000000000 6.3640696037406315

Ag Cl

8 16

Direct

0.6775433974514019 0.6199640988893074 0.4323383949795297

0.3224565725485954 0.3800359011106926 0.5676616350204728

0.8224566025485981 0.8800359011106926 0.9323383649795272

0.1775434724514047 0.1199640988893073 0.0676616200204751

0.3224629944094945 0.6199380615765012 0.0676897105974632

0.6775370055905050 0.3800619684234944 0.9323102934025375

0.1775370645905065 0.8800619384234988 0.5676897065974625

0.8224629944094950 0.1199380315765057 0.4323102934025301

0.8187354843957486 0.5778418320772228 0.0981697095023641

0.1812645006042537 0.4221581379227820 0.9018303204976313

0.6812645156042514 0.9221581679227772 0.5981696795023687

0.3187354843957486 0.0778418690772224 0.4018303204976313

0.1812918250214694 0.5778438516931936 0.4018465753007146

0.8187081449785280 0.4221561183068036 0.5981533946992903

0.3187081749785306 0.9221561483068064 0.9018466053007097

0.6812918550214720 0.0778438696931954 0.0981534396992903

0.4999830170577894 0.6604490805098870 0.7500098048010250

0.5000169829422104 0.3395509194901128 0.2499901801989780

0.0000169789422101 0.8395509194901130 0.2500098048010245

-0.0000169829422105 0.1604490215098860 0.7499901951989750

0.5000269528813522 0.1995766723169843 0.7499931809684337

0.4999730471186548 0.8004232676830182 0.2500068190315665

-0.0000269528813451 0.3004233276830158 0.2499931809684332

0.0000269438813480 0.6995767323169818 0.7500068190315663

## HP-AgF_2_ phonon-relaxed, 30 GPa, PBSEsol+U

HP-AgF2

1.00000000000000

5.6803389258198118 0.0881054654172212 0.0000000000000000

-0.1298324441294190 9.1065060576136148 0.0000000000000000

0.0000000000000000 0.0000000000000000 6.6889006620263407

Ag Cl

8 16

Direct

0.7028023770946179 0.6177616519071583 0.4127077529177761

0.2971975929053796 0.3822383480928351 0.5872922770822266

0.7971976229053821 0.8822383480928417 0.9127077229177734

0.2028024520946206 0.1177616519071649 0.0872922620822287

0.2962386792312722 0.6182885287812278 0.0879351963194325

0.7037613207687279 0.3817115012187677 0.9120648076805676

0.2037613797687292 0.8817114712187722 0.5879351923194324

0.7962386792312721 0.1182884987812323 0.4120648076805679

0.8543379089832502 0.6356464038390504 0.0665281123430938

0.1456620760167522 0.3643535661609543 0.9334719176569014

0.6456620910167498 0.8643535961609496 0.5665280823430986

0.3543379089832500 0.1356464408390498 0.4334719176569015

0.1446145836794180 0.6357510003728818 0.4339515329260596

0.8553853863205793 0.3642489696271156 0.5660484370739448

0.3553854163205819 0.8642489996271182 0.9339515629260552

0.6446146136794207 0.1357510183728835 0.0660484820739451

0.4999823940862287 0.5787156570760300 0.7502394363980397

0.5000176059137714 0.4212843429239699 0.2497605486019626

0.0000176019137710 0.9212843429239700 0.2502394363980396

-0.0000176059137714 0.0787155980760287 0.7497605636019603

0.4998101064699214 0.1509376554222613 0.7495125060283431

0.5001898935300860 0.8490622845777406 0.2504874939716570

0.0001898935300857 0.3490623445777386 0.2495125060283431

0.9998100974699168 0.6509377154222594 0.7504874939716569

## HP-AgF_2_ phonon-relaxed, 40 GPa, PBSEsol+U

HP-AgF2

1.00000000000000

5.5426674277221650 0.0874630901248473 0.0000000000000000

-0.1353017626369733 8.9466514405139907 0.0000000000000000

0.0000000000000000 0.0000000000000000 6.6245945166799389

Ag Cl

8 16

Direct

0.6985576987024171 0.6183961019490365 0.4114864863270590

0.3014422712975811 0.3816038980509638 0.5885135436729437

0.8014423012975829 0.8816038980509635 0.9114864563270563

0.1985577737024192 0.1183961019490364 0.0885135286729458

0.3008222561891277 0.6186497660971236 0.0888937751182288

0.6991777438108726 0.3813502639028712 0.9111062288817713

0.1991778028108737 0.8813502339028764 0.5888937711182287

0.8008222561891274 0.1186497360971286 0.4111062288817712

0.8522964704115671 0.6335600029596568 0.0674199059664690

0.1477035145884352 0.3664399670403481 0.9325801240335261

0.6477035295884329 0.8664399970403432 0.5674198759664739

0.3522964704115668 0.1335600399596562 0.4325801240335264

0.1471497954883870 0.6335753792917271 0.4328112945183643

0.8528501745116104 0.3664245907082699 0.5671886754816400

0.3528502045116134 0.8664246207082729 0.9328113245183600

0.6471498254883896 0.1335753972917293 0.0671887204816403

0.4999538399631683 0.5843582900106585 0.7502216893269948

0.5000461600368321 0.4156417099893423 0.2497782956730075

0.0000461560368311 0.9156417099893415 0.2502216893269948

-0.0000461600368317 0.0843582310106566 0.7497783106730052

0.4998963847627478 0.1497257686071451 0.7497709495917200

0.5001036152372597 0.8502741713928569 0.2502290504082804

0.0001036152372591 0.3502742313928550 0.2497709495917198

-0.0001036242372564 0.6497258286071430 0.7502290504082800
